# MCT4 is induced by metastasis-enhancing pathogenic mitochondrial NADH dehydrogenase gene mutations and can be a therapeutic target

**DOI:** 10.1038/s41598-021-92772-1

**Published:** 2021-06-25

**Authors:** Keizo Takenaga, Nobuko Koshikawa, Miho Akimoto, Yasutoshi Tatsumi, Jason Lin, Makiko Itami, Hiroki Nagase

**Affiliations:** 1 Laboratory of Cancer Genetics, Chiba Cancer Center Research Institute, 666-2 Nitona-cho, Chuoh-ku, Chiba, 260-8717 Japan; 2 Department of Biochemistry, Teikyo University School of Medicine, 2-11-1 Kaga, Itabashi-ku, Tokyo, 173-8605 Japan; 3 Laboratory of Oncogenomics, Chiba Cancer Center Research Institute, 666-2 Nitona-cho, Chuoh-ku, Chiba, 260-8717 Japan; 4 Department of Pathology, Chiba Cancer Center Hospital, 666-2 Nitona-cho, Chuoh-ku, Chiba, 260-8717 Japan

**Keywords:** Cancer, Cell biology, Molecular biology, Oncology

## Abstract

Pathogenic mitochondrial NADH dehydrogenase (*ND*) gene mutations enhance the invasion and metastasis of various cancer cells, and they are associated with metastasis in human non-small cell lung cancer (NSCLC). Moreover, monocarboxylate transporter 4 (MCT4) is overexpressed in solid cancers and plays a role in cancer cell proliferation and survival. Here, we report that MCT4 is exclusively expressed in mouse transmitochondrial cybrids with metastasis-enhancing pathogenic *ND6* mutations. A high level of MCT4 is also detected in human NSCLC cell lines and tissues predicted to carry pathogenic *ND* mutations and is associated with poor prognosis in NSCLC patients. MCT4 expression in the cell lines is suppressed by N-acetyl-L-cysteine. Phosphatidylinositol-3 kinase (PI3K), AMP-activated protein kinase (AMPK) and mechanistic target of rapamycin (mTOR) are involved in the regulation of MCT4 expression in the transmitochondrial cybrid cells. An MCT1/4 inhibitor effectively kills NSCLC cells with predicted pathogenic *ND* mutations, but an MCT1/2 inhibitor does not have the same effect. Thus, MCT4 expression is augmented by pathogenic *ND* mutations and could be a biomarker and a therapeutic target in pathogenic *ND* mutation-harbouring metastatic tumours.

## Introduction

It is well known that pathogenic mutations of mitochondrial DNA (mtDNA) are responsible for mitochondrial diseases such as Leber’s hereditary optic neuropathy (LHON), mitochondrial encephalomyopathy with lactic acidosis and stroke-like episodes (MELAS) and chronic progressive external ophthalmoplegia (CPEO)^1^. mtDNA mutations are also implicated in carcinogenesis^2^. In addition, we and others have reported that pathogenic mutations in the NADH dehydrogenase (*ND*) genes that cause complex I defects and deletion mutations in the nuclear DNA (nDNA)-coded mitochondrial complex I subunit genes enhance the invasion/metastasis of cancer cells^3–11^. Using transmitochondrial cybrids established from low-metastatic Lewis lung carcinoma cells and mouse fibrosarcoma cells, we reported that the G13997A missense mutation and 13981insC frame-shift mutation in *ND6* enhanced distant metastasis^3,4^. Further, the involvement of G13997A in the regulation of diabetes development, lymphoma formation and metastasis was demonstrated in transmitochondrial mice (mito-mice)^5^. C4859T and C4206T missense mutations in *ND2* conferred tumorigenic and metastatic potential to L929 cells^6^. We and others also demonstrated the enhancement of invasiveness and metastasis of various human cancer cell lines with *ND* gene mutations, including G10398A in *ND3*, G13289A in *ND5*, loss of *NDUFV1* or loss of *NDUFB9*^7–12^. *ND6* missense and nonsense mutations detected in human lung cancer tissues enhanced the in vitro invasiveness of lung cancer cells^11^. Furthermore, the frequency of predicted pathogenic *ND* gene missense mutations and nonsense mutations was significantly associated with distant metastasis in human NSCLC patients^4^. An interesting aspect of these reports is that most of the pathogenic mutations that enhance invasion/metastasis reside in *ND* genes. These results indicate the importance of verifying pathogenic *ND* mutations in cancer tissues to predict metastasis. Therefore, finding a biomarker that is associated with pathogenic *ND* mutations and can be easily examined by routine examination is highly relevant to the selection of appropriate treatment modalities and for prognosis prediction.

The monocarboxylate transporter (MCT) family comprises 14 members, and its members are encoded by the solute carrier family 16 (*SLC16*) gene family. They transport pyruvate, L-lactate and short-chain fatty acids, monocarboxylate drugs and other substrates across the cell membrane in a wide variety of tissues. Among them, MCT1-4 are demonstrated to be proton-coupled lactate transporters. MCT1 is widely expressed, whereas MCT4 tends to be restricted to glycolytic tissues such as muscle and cancers^13–15^. MCT2 and MCT3 are not well characterized. Notably, MCT4 expression has been shown to be regulated by hypoxia-inducible factor-1 (HIF-1), which upregulates the gene expression of many glycolytic enzymes and other hypoxia-related genes, such as those involved in angiogenic and malignant behaviour of cancer cells^16^. However, the precise mechanisms by which MCT4 expression is regulated are largely unknown. Recent studies have revealed substrates for other MCTs, but their roles in cancers are unknown. MCT1, MCT3 and MCT4 interact with the transmembrane glycoprotein CD147 (also known as basigin or EMMPRIN), whereas MCT2 forms a complex with gp70 (also known as embigin)^13–15^. CD147 acts as a cochaperone for the trafficking of MCT1, 3 and 4 to the plasma membrane, and its interaction with MCTs is required for their proper function^17^.

One of the characteristic features of cancer cells is the increased uptake of glucose and enhanced glycolysis that metabolizes glucose to lactate even in the presence of functional mitochondria, which is known as the “Warburg effect”^18,19^. Cancer cells with malfunctioning mitochondria caused by either pathogenic mtDNA mutations or nDNA-coded respiratory chain subunit gene mutations also exhibit enhanced glycolysis, resulting in the production of energy and a larger amount of lactate than what is observed in cells with normal mitochondria^20^. Under hypoxic conditions, cancer cells express HIF-1 and rewire their glycolytic metabolism to proliferate and survive^21^. Thus, intracellular lactate that is produced has to be exported out of cancer cells to prevent them from intracellular acidification and cell death. To this end, MCTs play important roles, and MCT1 and MCT4 have been shown to be overexpressed in a number of cancers, including glioblastoma, colon cancer, breast cancer, prostate cancer, clear cell renal cell carcinoma, oesophageal squamous carcinoma pancreatic cancer and non-small cell lung carcinoma (NSCLC)^13–15,22^. Furthermore, they have been proposed to be diagnostic or prognostic markers in cancer patients^23–29^. Small interfering RNA (siRNA)-mediated knockdown of MCT1 or MCT4 or treatment of cells with MCT inhibitors suppressed growth and invasiveness of cancer cells^30–34^. As such, MCTs are thought to be potential therapeutic targets^30,35–37^.

Cancer cells with metastasis-enhancing pathogenic *ND* mutations show complex I defects, so they produce a large amount of lactate. Hence, they would be expected to express a higher level of MCTs than cancer cells with wild type mtDNA; however, this has not been investigated. Additionally, it remains to be examined whether there is a relationship between pathogenic *ND* mutations and the expression of MCTs and metastasis. We sought to solve these issues, and we report here that metastasis-enhancing *ND* mutations specifically induce MCT4 but no other MCTs, as shown in cybrid cells established from mtDNA-less ρ^0^P29 cells (derived from low-metastatic Lewis lung carcinoma P29 cells^3^) and human NSCLC cells and tissues. The results indicate that MCT4 expression could be a biomarker of pathogenic *ND* mutation-mediated metastasis and a potential metabolic target for the killing of cancer cells with such *ND* mutations.

## Results

### MCT4 expression is correlated with the metastatic potential of the P29 cybrids

**Figure 1:**
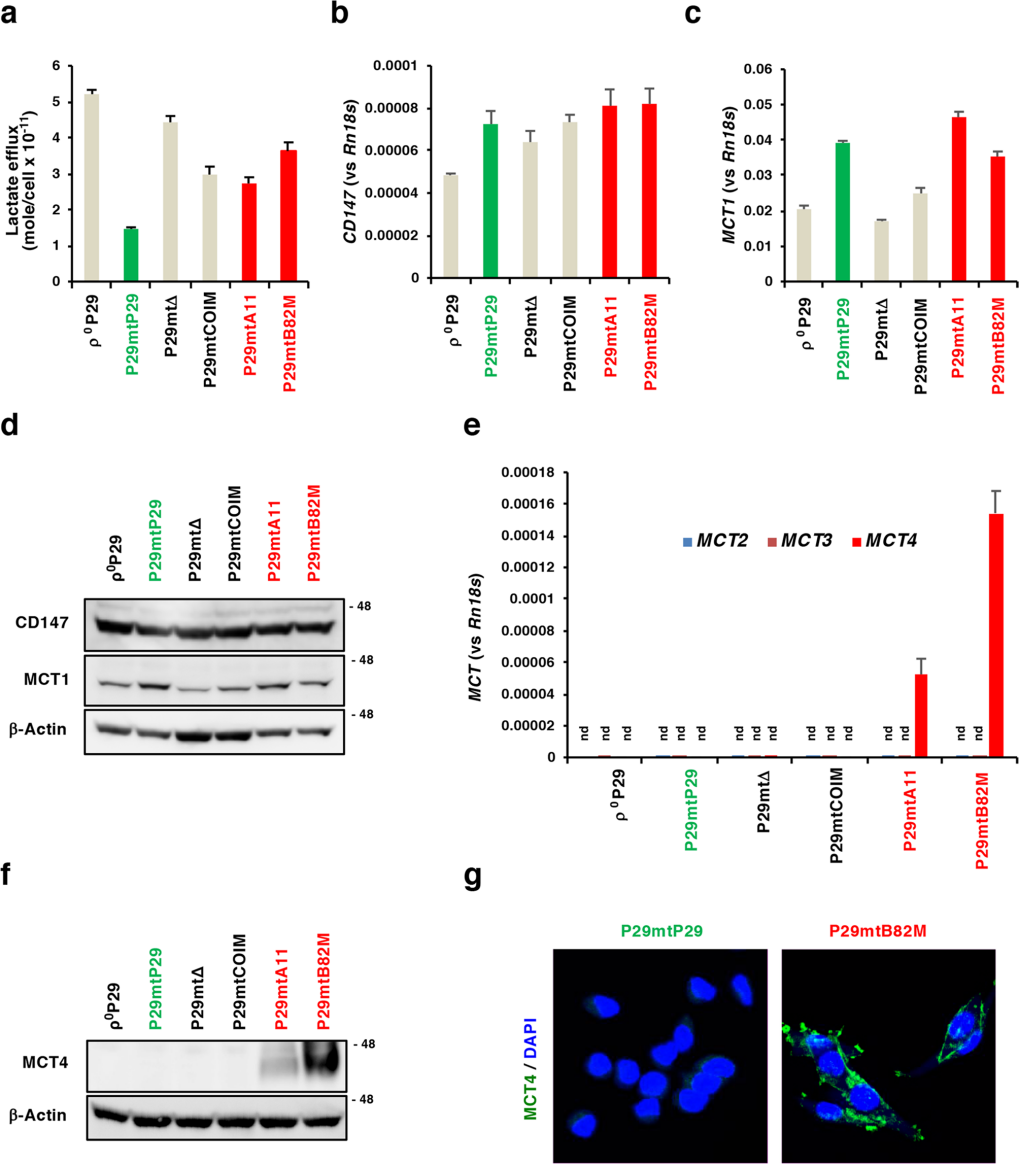
Lactate efflux and the expression of lactate transporter MCTs. (**a**) Lactate efflux by P29 cybrids. Error bar: SD. (**b**) RT-qPCR analysis of CD147 mRNA expression in P29 cybrids. Error bar: SD. (**c**) RT-qPCR analysis of MCT1 mRNA expression in P29 cybrids. Error bar: SD. (**d**) Western blot analysis of the expression of CD147 and MCT1 in P29 cybrids. β-Actin was used as a loading control. (**e**) RT-qPCR analysis of the expression of MCT2, MCT3 and MCT4 mRNA in P29 cybrids. nd: not detected. Error bar: SD. (**f**) Western blot analysis of the expression of MCT4 in P29 cybrids. β-Actin was used as a loading control. (**g**) Immunofluorescent staining of MCT4 in P29mtP29 and P29mtB82M cells. Uncropped Western blots images are shown in Supplementary Fig. S12.

We compared the tumorigenicity and metastatic potential of a series of P29 cybrids that have the same nDNA but different mtDNA (Table 1). ρ^0^P29 cells (mtDNA-less) and P29mtΔ cells (mtDNA with a 4696 bp deletion) were nontumorigenic, while P29mtP29 (wild-type mtDNA), P29mtCOIM (mtDNA with a *COI* T6589C missense mutation), P29mtA11 (mtDNA with an *ND6* G13997A missense mutation) and P29mtB82M (mtDNA with an *ND6* 13891insC frame-shift mutation) cells were tumorigenic. The order of spontaneous metastatic ability among tumorigenic cybrids was P29mtCOIM = P29mtP29 < P29mtA11 < < P29mtB82M (Table 1). To see if any of these cybrids exhibited the metabolic phenotype that is well correlated with metastatic ability, we first compared the level of glycolytic activity. For this, we examined glucose uptake, expression levels of glycolytic enzymes, lactate production and their related gene expression. The results showed that neither glucose uptake nor glucose transporter 1 (*Glut1*) expression was associated with their respective metastatic ability (Supplementary Fig. S1). The expression level of each glycolytic enzyme also did not show any association with metastatic ability (Supplementary Fig. S1). On the other hand, the order of lactate production among the cybrids was P29mtP29 < P29mtCOIM = P29mtA11 < P29mtB82M < P29mtΔ < ρ^0^P29, i.e., the more severe the respiratory chain defect was, the larger the amount of lactate produced by the cells was (Fig. 1a). However, lactate production was also not correlated with metastatic ability among the tumorigenic cybrids. We next examined the expression of lactate transporters MCT1-4 and the cochaperone CD147 in the cybrids. All the cybrids expressed CD147 and MCT1 at both the mRNA and protein levels, but they did not express MCT2 or MCT3 (Fig. 1b–e). MCT1 expression did not necessarily correspond to the degree of lactate production. Moreover, there was no association between the expression level of CD147 or MCT1 and metastatic ability (Fig. 1b,c and Table 1). Remarkably, on the other hand, it appeared that *MCT4* mRNA was exclusively detected in P29mtA11 and P29mtB82M cells, which was confirmed by Western blotting and immunofluorescent staining, and most importantly, the expression level of MCT4 was proportional to the metastatic ability (Fig. 1e–g and Table 1). We also examined the expression of several other MCTs in P29 cybrids and found that *MCT7*, a transporter of ketone bodies^15^, was more highly expressed in P29mtA11 and P29mtB82M than it was in other cybrids (Supplementary Fig. S2). However, because *MCT7* expression was not associated with pathogenic *ND* mutations in NSCLC cell lines (as described below), we did not investigate *MCT7* in detail in this study. To further confirm MCT4 expression in P29mtP29 and P29mtB82M cells and to seek other genes that were expressed in correlation with metastatic potential, the cells were subjected to RNA-seq analysis, and differences in gene expression profiles between the different cells were analysed (Supplementary Fig. S3). While the expression profiles between the two remain largely closely correlated, some genes deviated from this trend; coinciding with the above results, we found *MCT4* (*Slc16a3*) to be among the top 5 most upregulated genes in P29mtB82M cells. For the purpose of discussion, the other 4 most upregulated genes included *Col6a3* (collagen type VI α3 chain), *Col5a3* (collagen type V α3 chain), *Krt8* (keratin 8) and *Gatm* (mitochondrial glycine amidinotransferase), but there was no clear association between their expression levels and metastatic potential. Gene set enrichment analysis revealed no particularly enriched pathways among the top upregulated genes, suggesting that the mutation did not cause any concerted effect in the activation of particular gene clusters but instead triggered highly specific functional alterations in these genes. It should be noted that keratin 8, which has been shown to be overexpressed in various human cancers and is associated with poor prognoses in lung adenocarcinomas^38^, was expressed only in cybrid cells with *ND6* mutations (P29mtA11 and P29mtB82M cells) (Supplementary Fig. S3). The lack of concerted pathway enrichment led us to explore how individual pathways responded to the effect of the 13891insC mutation in *ND6* by evaluating the mean changes in expression in genes involved in these clusters. Among all the mouse KEGG pathways, we evaluated the arithmetic mean of fold changes and observed the upregulation of amino acid biosynthesis pathways and downregulation of glycosaminoglycan and glycosphingolipid biosynthesis pathways in P29mtB82M cells (Supplementary Fig. S4).

**Table 1:** Tumourigenicity and metastatic potential of the P29 cybrids used in this study.

Cybrid	mtDNA	Tumourigenicity	No. of pulmonary metastasis		
		Tumour take/No. of mice tested	No. of mice with metastatic foci / No. of mice tested	Median (Range)	*P* value* (vs P29mtP29)
ρ^0^P29	None	0/6	nd	–	
P29mtP29	Wild-type	8/8	3/6	0.5 (0–4)	
P29mtΔ	4696 bp deletion	0/6	nd	–	
P29mtCOIM	*COI* G13997A	13/14	1/7	0 (0–1)	ns
P29mtA11	*ND6* G13997A	8/8	7/7	2 (1–9)	0.0329
P29mtB82M	*ND6* 13891insC	8/8	7/7	38 (16–87)	0.0059

nd, not detected; ns, not significant.

*Mann–Whitney U test.

### MCT4 expression is involved in the invasiveness of P29mtB82M cells

**Figure 2:**
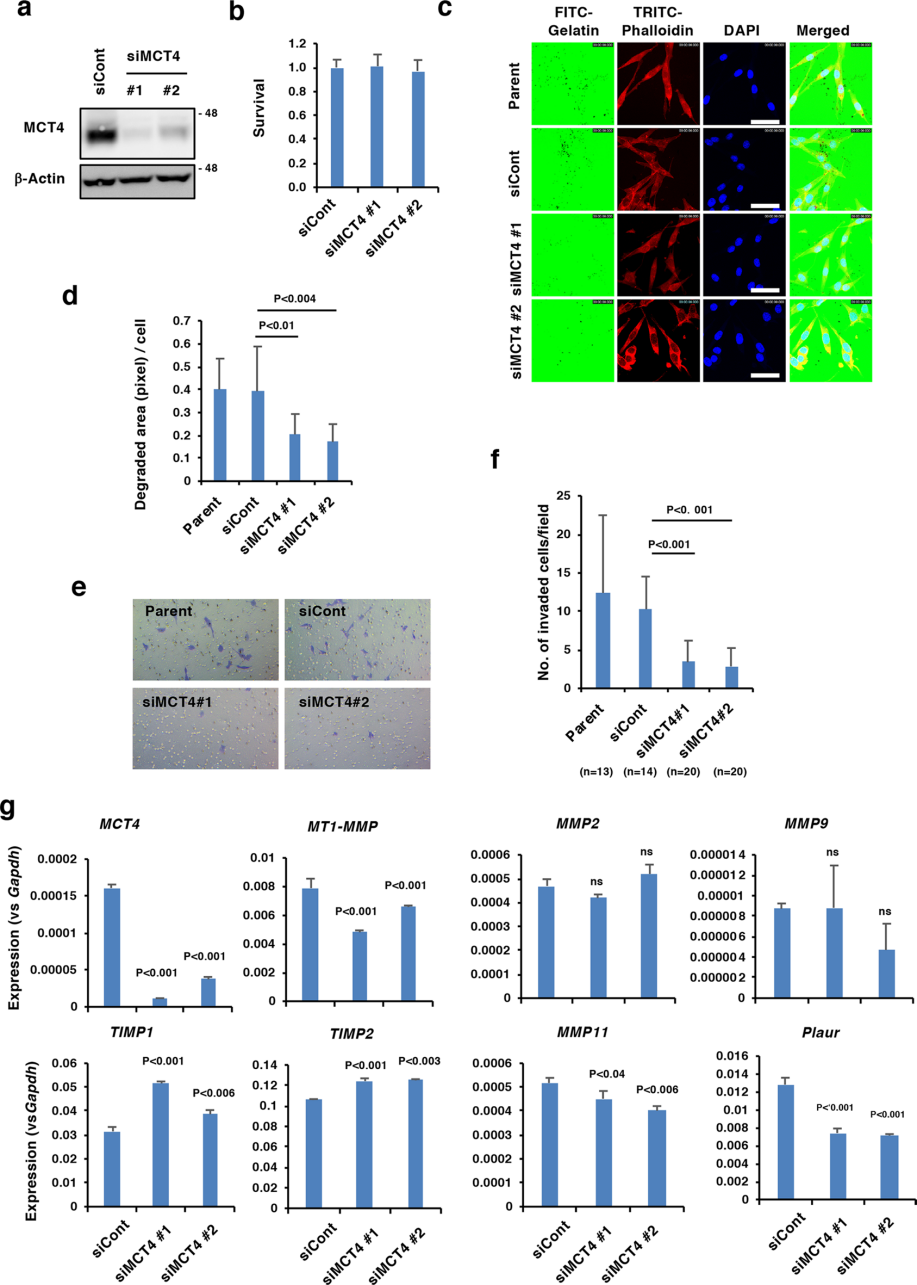
Effect of MCT4 knockdown on the invasiveness of P29mtB82M cells. (**a**) Western blot analysis of the expression of MCT4 in P29mtB82M cells transfected with a control siRNA (siCont) or an MCT4 siRNA (siMCT4 #1 and #2). β-Actin was used as a loading control. Uncropped Western blot images are shown in Supplementary Fig. S12. (**b**) Proliferation of P29mtB82M cells transfected with a control siRNA (siCont) or an MCT4 siRNA (siMCT4 #1 and #2), as measured by WST assay. The cells were cultured for 2 days. Error bar: SD. (**c**) Gelatine invadopodia assay. Images showing the degradation of FITC-labelled gelatine at the invadopodia of P29mtB82M cells transfected with a control siRNA (siCont) or an MCT4 siRNA (siMCT4 #1 and #2). Bar. 20 μm. (**d**) Quantitation of the degraded area/cell in the invadopodia assay. Error bar: SD. (**e**) Matrigel invasion assay. Images showing invasion of P29mtB82M cells transfected with a control siRNA (siCont) or an MCT4 siRNA (siMCT4 #1 and #2). (**f**) Quantitation of the number of invaded cells. Error bar: SD. (**g**) RT-qPCR analysis of the expression of MCT4 and matrix degradation-related genes in P29mtB82M cells transfected with a control siRNA (siCont) or MCT4 siRNAs (siMCT4 #1 and #2). Error bar: SD.

To investigate whether MCT4 plays any role in the invasiveness of P29mtB82M cells, we knocked down MCT4 expression in the cells with two different siRNAs (siMCT4 #1 and #2) (Fig. 2a). The results showed that knockdown of MCT4 did not affect cell proliferation (Fig. 2b). On the other hand, both siRNAs suppressed invadopodia formation, as assessed by a gelatine invadopodia formation assay (Fig. 2c,d), and invasiveness, as shown by a Matrigel invasion assay (Fig. 2e,f). Consistent with these results, the expression levels of membrane type 1-matrix metalloproteinase (*MT1-MMP*), *MMP11* and plasminogen activator urokinase receptor (*Plaur*) were slightly but significantly downregulated by the siRNAs. Although the siRNAs did not affect the expression of *MMP2* and *MMP9*, they upregulated the expression of the corresponding tissue inhibitors of metalloprorteinases-2 (*TIMP2*) and *TIMP1* (Fig. 2g). These results indicate that MCT4 not only functions as a lactate transporter but also is involved in the regulation of invasiveness in P29mtB82M cells. We investigated the possible role of MCT4 in epithelial-mesenchymal transition (EMT) by examining the expressions of EMT-related marker genes (*Snai1*, *Snai2*, *Twist1*, *Twist2*, *Zeb1*, *Zeb2*, *Vim* and *Cdh1*) in siMCT4-transfected P29mtB82M cells, and while results suggested possible alterations in the expression of these genes, we found no consistent changes linked to the downregulation of MCT4 expression (Supplementary Fig. S5). These results may exclude the possibility of MCT4 involvement in EMT although it cannot be ruled out that EMT is inhibited in siMCT4 cells via post-transcriptional regulation of EMT regulators.

### Correlation of ND mutations and MCT4 expression in human NSCLC cell lines

**Figure 3:**
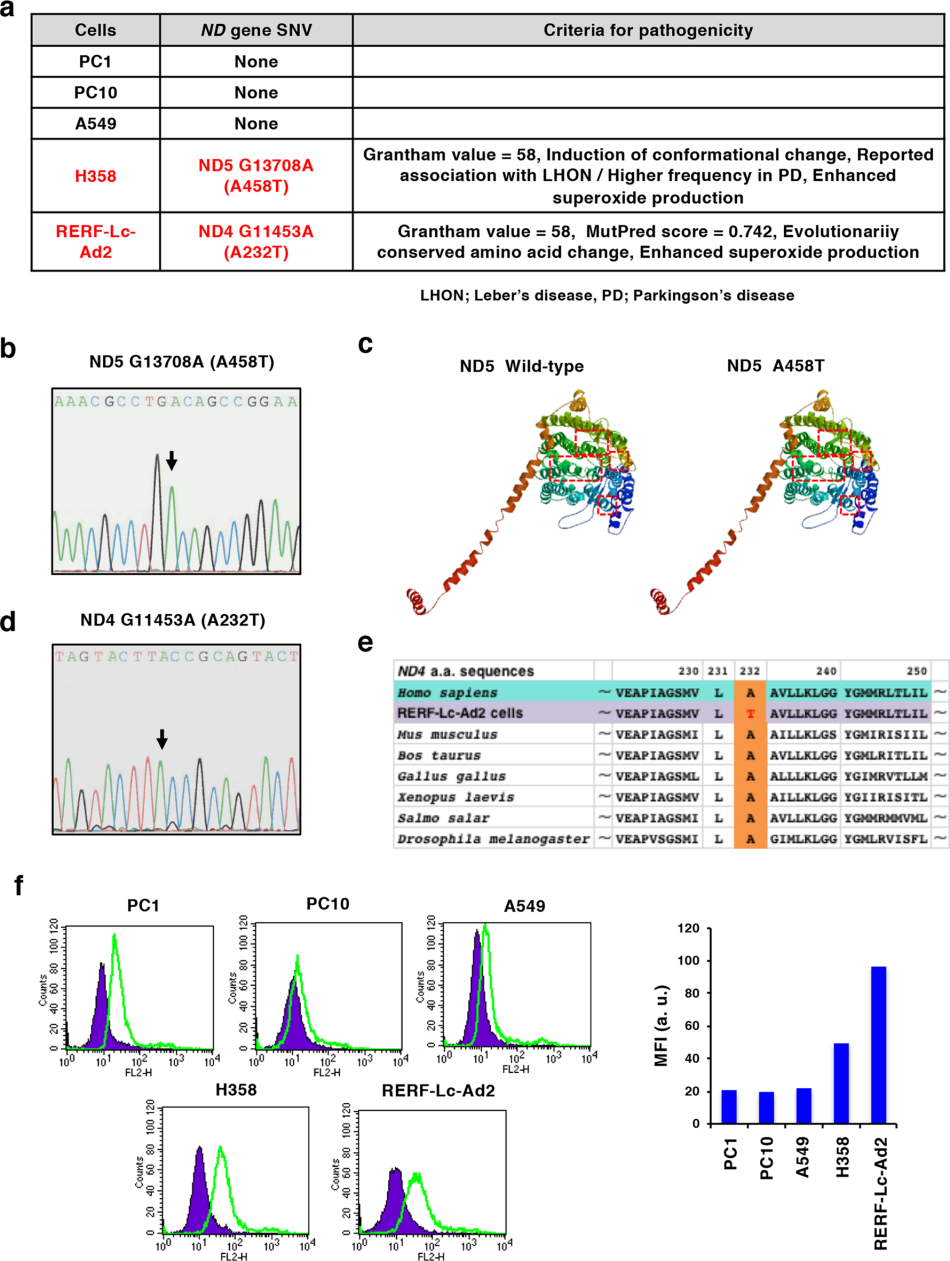
Predicted pathogenic ND mutations found in H358 and RERF-Lc-Ad2 cells. (**a**) Predicted pathogenic SNVs found in NSCLC cells and the criteria for the prediction of pathogenicity. Refer to MITOMAP (https://www.mitomap.org/MITOMAP) for information about disease associations. (**b**) Electropherogram showing the *ND5* G13708A (A453T) SNV. (**c**) Conformational change of the protein caused by *ND5* G13708A (A453T) SNV, as predicted by SWISS-MODEL (https://swissmodel.expasy.org). Rectangles with a red dashed border indicate the areas containing the conformational change caused by the SNV. (**d**) Electropherogram showing *ND4* G11453A (A232T) SNV. (**e**) Evolutionary conservation of the alanine residue. (**f**) Mitochondrial ROS production in NSCLC cell lines. ROS production was examined by MitoSOX-Red staining; mean fluorescence intensity (MFI) were expressed as arbitrary units (a. u.).

**Figure 4:**
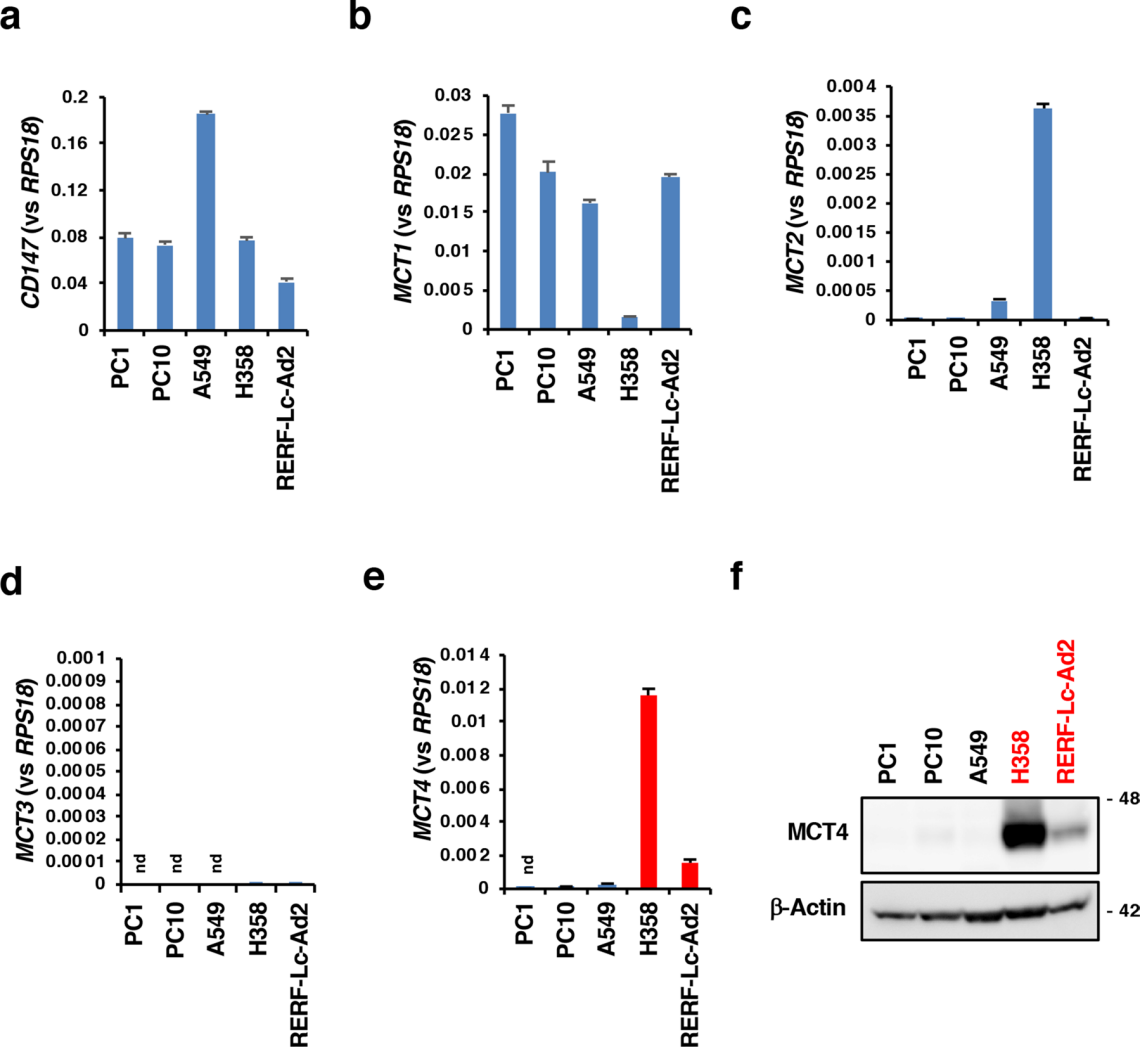
MCT expression in NSCLC cell lines. (**a**–**e**) RT-qPCR analysis of the expression of CD147 and MCT1-4. nd: not detected. (**f**) Western blot analysis of MCT4 expression. β-Actin was used as a loading control. Uncropped Western blot images are shown in Supplementary Fig. S12.

To investigate the relationship between *ND* gene mutations and MCT4 expression in NSCLC cell lines, we searched for *ND* gene mutations in several NSCLC cell lines (PC1, PC10, A549, H358 and RERF-Lc-Ad2). According to the criteria we used in the previous report to predict the pathogenicity of certain *ND* mutations [Grantham value > 50, MutPred score > 0.7, evolutionary conservation of the original amino acid, conformational change of the protein predicted by SWISS-MODEL, the association with mitochondrial diseases reported in the MITOMAP database and mitochondrial reactive oxygen species (mtROS) overproduction]^4^, we extracted homoplasmic *ND5* G13708A (A458T) in H358 cells and homoplasmic *ND4* G11453A (A232T) in RERF-Lc-Ad2 cells as predicted pathogenic mutations (Fig. 3a and Supplementary Table S1). The *ND5* G13708A mutation was predicted to result in conformational changes in the protein, was reported to be associated with Leber’s disease and Parkinson’s disease, and caused mtROS overproduction (Fig. 3a–c, f and Supplementary Table S2). The *ND4* G11453A mutation had a high MutPred score, resulted in the change in conserved amino acids and caused mtROS overproduction (Fig. 3a, d–f and Supplementary Table S2). H358 cells also had a missense mutation, *ND1* T4216C (Y304H), that was not predicted to be pathogenic but showed a high Grantham value and high disease associations (Supplementary Tables S1 and S2). No candidates of pathogenic *ND* mutations were found in other cell lines based on our criteria (Fig. 3a, Supplementary Tables S1 and S2). We examined the expression of CD147 and MCTs in these cell lines. CD147 was detected in all the cell lines (Fig. 4a). H358 cells expressed a lower amount of MCT1 than other cell lines, but they expressed higher levels of MCT2 (Fig. 4b,c). MCT3 was not expressed in any of the cell lines (Fig. 4d). Interestingly, as in the case with P29 cybrids, we only detected the expression of *MCT4* mRNA in H358 and RERF-Lc-Ad2 cells (Fig. 4e). Higher expression of MCT4 in H358 cells than in RERF-Lc-Ad2 cells relative to other tested cells was also confirmed at the protein level (Fig. 4f). Although MCT6, the substrates of which are bumetanide, nateglinide, probenecid, and prostaglandin F2α^39^, was also expressed in H358 and RERF-Lc-Ad2 cells (Supplementary Fig. S6), we did not examine its role in this study.

### MCT4 expression in NSCLC tissues and its correlation with prognosis

**Figure 5:**
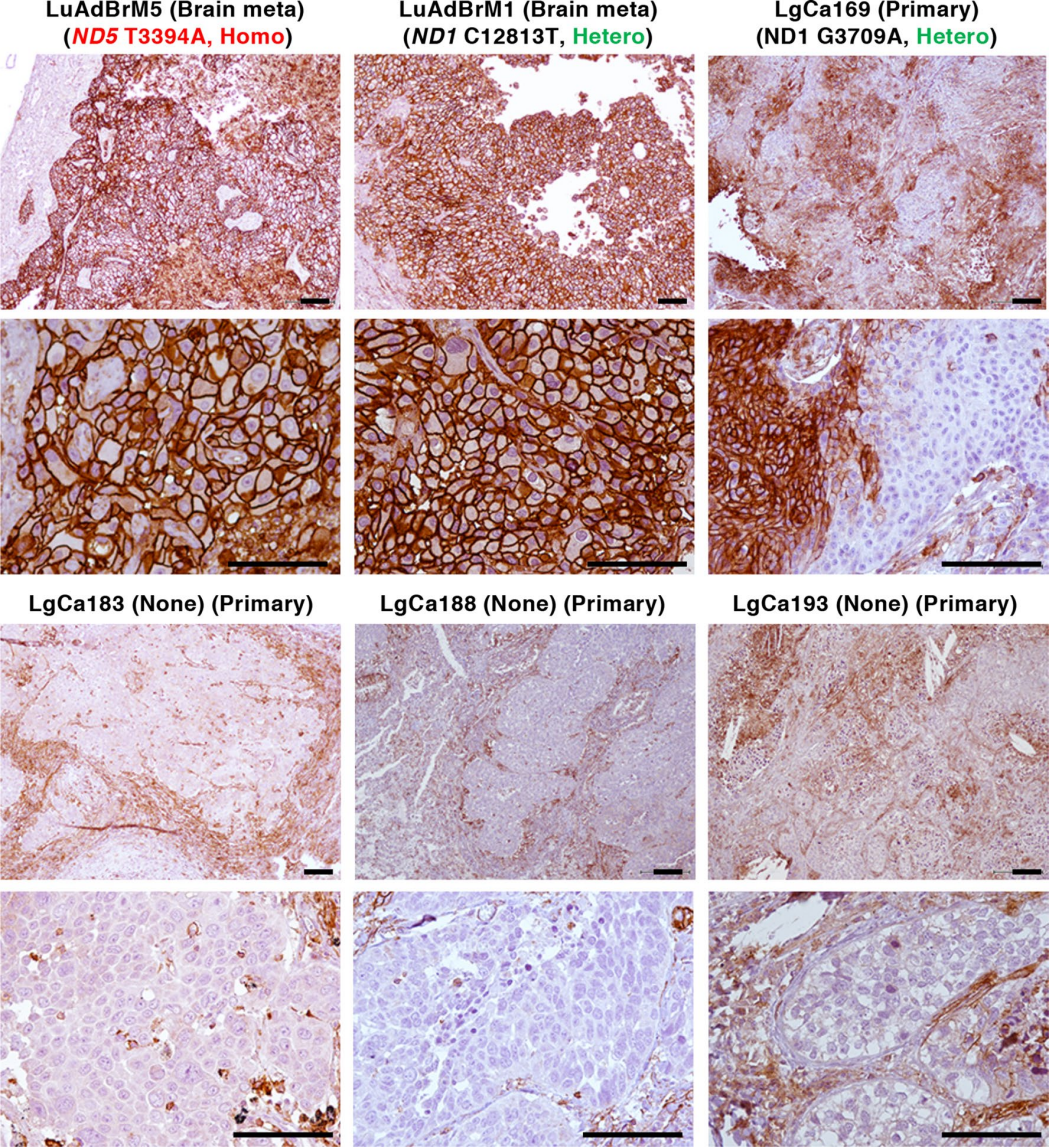
IHC analysis of MCT4 expression in NSCLC tissues. NSCLC tissues harbouring predicted pathogenic ND mutations such as homoplasmy (LuAdBrM5) or heteroplasmy (or heterogeneous state) (LuAdBrM1 and LgCa169) and NSCLC tissues having no pathogenic ND mutation (LgCa183, LgCa188 and LgCa193) were immunostained for MCT4 using a monoclonal MCT4 antibody. LuAdBrM5 and LuAdBrM1 tissues were strongly positive for MCT4 on cell membranes. LgCa169 tissues contain both MCT4-positive and MCT4-negative regions, which might represent cancer cells with and without the mutation, respectively. Cancer cells in LgCa183, LgC188 and LgCa193 tissues were negative for MCT4, but stromal cells were positive. Bar: 100 μm.

Based on the above observations, we immunostained primary NSCLC tissues (designated as P in Table 2) and metastatic brain tissues (designated as M) with or without predicted pathogenic *ND* mutations^4^ for MCT4 using a monoclonal antibody. The results showed that LuAdBrM5 tissues that have homoplasmic predicted pathogenic mtDNA mutations^4^ exhibited strong MCT4 expression on the membrane of cancer cells (Fig. 5 and Table 2). LuAdBrM1 and LgCa169 tissues have predicted heteroplasmic pathogenic *ND* mutations or are in a heterogeneous state, meaning a mixture of cancer cells with the mutation, those without the mutation and/or host-derived stromal cells with wild-type mtDNA, and they showed different features (Fig. 5 and Table 2). In the case of LuAdBrM1, all cancer cells were strongly positive for MCT4, indicating genuine heteroplasmy. In the case of LgCa169, the tissue contained MCT4-positive and MCT4-negative cancer cells with relatively clear boundaries. On the other hand, cancer regions in NSCLC tissues without predicted pathogenic *ND* mutations (LgCa183, LgCa188 and LgCa193) exhibited weak MCT4 expression (Fig. 5 and Table 2). In almost all cases, stromal cells, including immune cells, exhibited MCT4 staining. A similar tendency was observed in other NSCLC tissues with or without predicted pathogenic *ND* mutations; the exception was LgCa157 tissues, in which strong MCT4 staining was observed irrespective of the absence of pathogenic *ND* mutations (Supplementary Fig. S7). In the case of LgCa179, MCT4 staining was observed in focal regions, implicating that MCT4-positive cancer cells were localized to hypoxic regions (Supplementary Fig. S7). To date, 8 out of 9 NSCLC tissues with predicted pathogenic *ND* mutations contained strongly MCT4-positive cancer cells (IHC score > 3 +), while only 1 out of 8 tissues without pathogenic *ND* mutations contained strongly positive cells (*P* < 0.05, χ^2^ test with Yates’ correction) (Table 2). It should be noted that 6 out of 8 NSCLC tissues with predicted pathogenic *ND* mutations and high MCT4 expression were metastatic samples. Thus, although the number of specimens was small, there was a trend towards an association between the presence of pathogenic *ND* mutations, the level of MCT4 expression and metastasis in NSCLC tissues.

**Table 2:** MCT4 expression in NSCLC tissues with or without predicted pathogenic mutations.

Patient ID	Histology	P/M	ND gene	Mutation	Amino acid change	Evolutionary conservation	MutPred score	Disease association	Homo/ Heterogen/ Hetero	IHC score
LuAdBrM5	Adenocarcinoma	M	ND1	**T3394C**	Y30H	Conserved	0.783	LHON/Diabetes/ CPT deficiency/ High altitude adaptation	Homo	** + + + + **
LuAdBrM8	Adenocarcinoma	M	ND1	**T3394C**	Y30H	Conserved	0.783	LHON/Diabetes/ CPT deficiency/ High altitude adaptation	Homo	** + + + **
LuAdBrM13	Adenocarcinoma	M	ND1	**T3394C**	Y30H	Conserved	0.783	LHON/Diabetes/ CPT deficiency/ High altitude adaptation	Homo	** + + + **
LgCa196	Adenocarcinoma	P	ND1	**C3497T**	A64V		0.413	LHON	Homo	** + + + **
LuAdBrM2	Adenocarcinoma	M	ND1	**C3497T**	A64V		0.413	LHON	Homo	** + + + + **
LuPoBrM1	Adenocarcinoma	M	ND1	**C3497T**	A64V		0.413	LHON	Homo	** + + + **
LgCa169	Squamous cell carcinoma	P	ND1	**H3709A**	A135T	Conserved	0.774		Heterogen or Hetero	** − and + + + + **
LuAdBrM1	Adenocarcinoma	M	ND5	**C12813A**	Y159stop				Heterogen or Hetero	** + + + + **
LgCa173	Squamous cell carcinoma	P	ND5	**G13103A**	G256E	Conserved	0.834		Heterogen or Hetero	** + + **
LgCa157	Squamous cell carcinoma	P		** None**						** + + + **
LgCa179	Adenocarcinoma	P		** None**						** + + (local)**
LgCa183	Squamous cell carcinoma	P		** None**						** + **
LgCa188	Squamous cell carcinoma	P		** None**						** + **
LgCa193	Squamous cell carcinoma	P		** None**						** + **
LgCa202	Adenocarcinoma	P		** None**						** + **
LgCa175	Adenocarcinoma	P		** None**						** + **
LgCa195	Bronchioalveolar carcinoma	P		** None**						** + **

Homo/Heterogen/Hetero: Homoplasmy/Heterogeneous/Heteroplasmy. Heterogeneous means a mixture of cancer cells with the mutation, those without the mutation and/or host-derived stromal cells with wild-type mtDNA.

P/M, Primary tumour/Metastases.

To examine the association between MCT4 expression and prognosis in NSCLC patients, we searched the public prognosis databases PROGgeneV2 (http://watson.compbio.iupui.edu/chirayu/proggene/database/?url=proggene) and Oncomine (https://www.oncomine.org/resource/login.html). The datasets showed that the patients with a higher MCT4 expression showed worse overall survival (OS) and relapse-free survival (RFS) (Supplementary Fig. S8). Higher MCT4 expression in cancer tissues at more advanced stages and a clear correlation between higher MCT4 expression and poorer prognosis were also observed in patients with lung adenocarcinoma (Supplementary Fig. S8).

### N-Acetylcysteine (NAC) suppresses MCT4 expression independently of ROS

**Figure 6:**
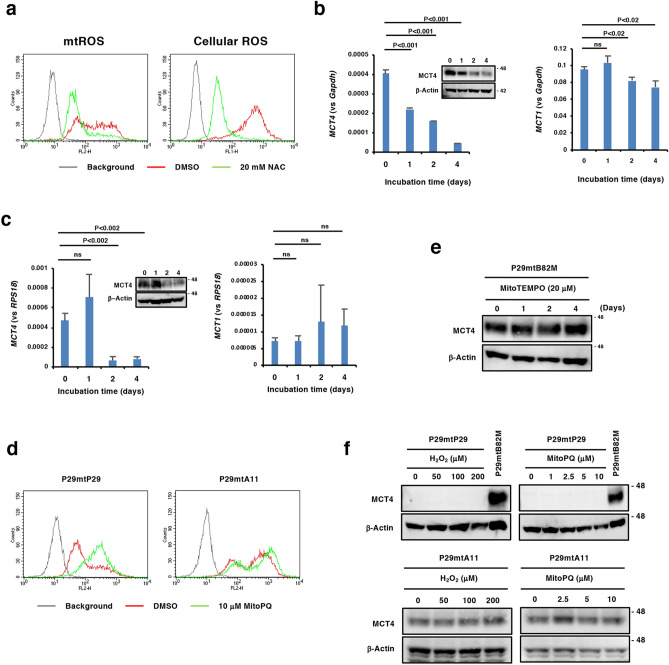
NAC suppresses MCT4 expression. (**a**) Effect of NAC on ROS generation in P29mtB82 cells by FACS analysis. Cells were treated with 20 mM NAC for 24 h, and levels of mtROS and cellular ROS were detected by MitoSOX Red and DCF-DA, respectively. (**b**, **c**) RT-qPCR and Western blot analyses of MCT4 and MCT1 expressions in P29mtB82M (**b**) and H358 cells (**c**), both of which were treated with 20 mM NAC for the indicated times. (**d**) FACS analysis of the effect of MitoPQ on mtROS generation in P29mtP29 and P29mtA11 cells. The cells were treated with 10 µM MitoPQ for 24 h. mtROS were detected by MitoSOX Red. (**e**) Effect of MitoTEMPO on MCT4 expression in P29mtB82M cells by Western blotting. The cells were treated with 20 µM MitoTEMPO for the indicated times. (**f**) Effect of H_2_O_2_ and MitoPQ. P29mtP29 and P29mtA11 cells were treated with H_2_O_2_ or MitoPQ at various concentrations for 3 and 2 days, respectively. Lysates from P29mtB82M cells were used as a positive control. β-Actin was used as a loading control. Uncropped Western blot images are available as Supplementary Fig. S12.

Because we previously reported that the ROS scavenger NAC suppressed the metastatic ability of cybrids with pathogenic *ND6* mutations^3^, we examined the effect of NAC on MCT4 expression in P29mtB82M cells and H358 cells. Our results revealed significant NAC-mediated suppression of mtROS and cellular ROS generation in P29mtB82M cells (Fig. 6a). NAC inhibited MCT4 expression at both the mRNA and protein levels relative to those of MCT1 in the cells (Fig. 6b). Similarly, MCT4 expression was suppressed by NAC at both the mRNA and protein levels in H358 cells, but a similar effect was not observed regarding MCT1 expression (Fig. 6c). To examine whether ROS production is attributed to the increase in MCT4 expression, we treated P29mtB82M cells with the mitochondria-targeting antioxidant MitoTEMPO, and we treated P29mtP29 and P29mtA11 cells with hydrogen peroxide and the mitochondria-targeting redox cycler MitoPQ, which produces superoxide by redox cycling at the flavin site of complex I. We confirmed the ability of MitoPQ to stimulate mtROS generation (Fig. 6d). MitoTEMPO did not inhibit MCT4 expression in P29mtB82M cells (Fig. 6e), and both hydrogen peroxide and MitoPQ failed to induce MCT4 expression in both P29mtP29 and P29mtA11 cells (Fig. 6f). These results indicate that MCT4 expression is ROS-independent, that the effect of NAC is not due to its antioxidant activity, and that enhanced mtROS generation caused by the *ND6* gene mutation is not a trigger of MCT4 expression.

### MCT4 expression is HIF-1-independent in P29mtB82 and H358 cells

We investigated the mechanisms by which MCT4 expression was induced in P29mtB82M cells. First, we examined the involvement of HIF-1. However, neither the HIF-1α inducers deferoxamine (DFO) and CoCl_2_ nor the HIF-1 inhibitors TX-402 and 2-methoxyestradiol (2-ME)^40,41^ affected MCT4 expression in P29mtP29 cells and P29mtB82M cells, respectively (Supplementary Fig. S9). Furthermore, although HIF-1α was detected at low levels in P29mtA11 and P29mtB82M cells relative to its levels in P29mtP29 cells, there was no association between the expression level of HIF-1α and that of MCT4 (Supplementary Fig. S9). DFO actually induced HIF-1α in P29mtP29 cells but did not induce MCT4 expression. HIF-1α was also not detected in the NSCLC cell lines (Supplementary Fig. S9). Thus, we concluded that HIF-1 is not responsible for MCT4 expression in cells with *ND* mutations.

### Signalling pathways involved in the enhancement of MCT4 expression in P29mtB82M and H358 cells

**Figure 7:**
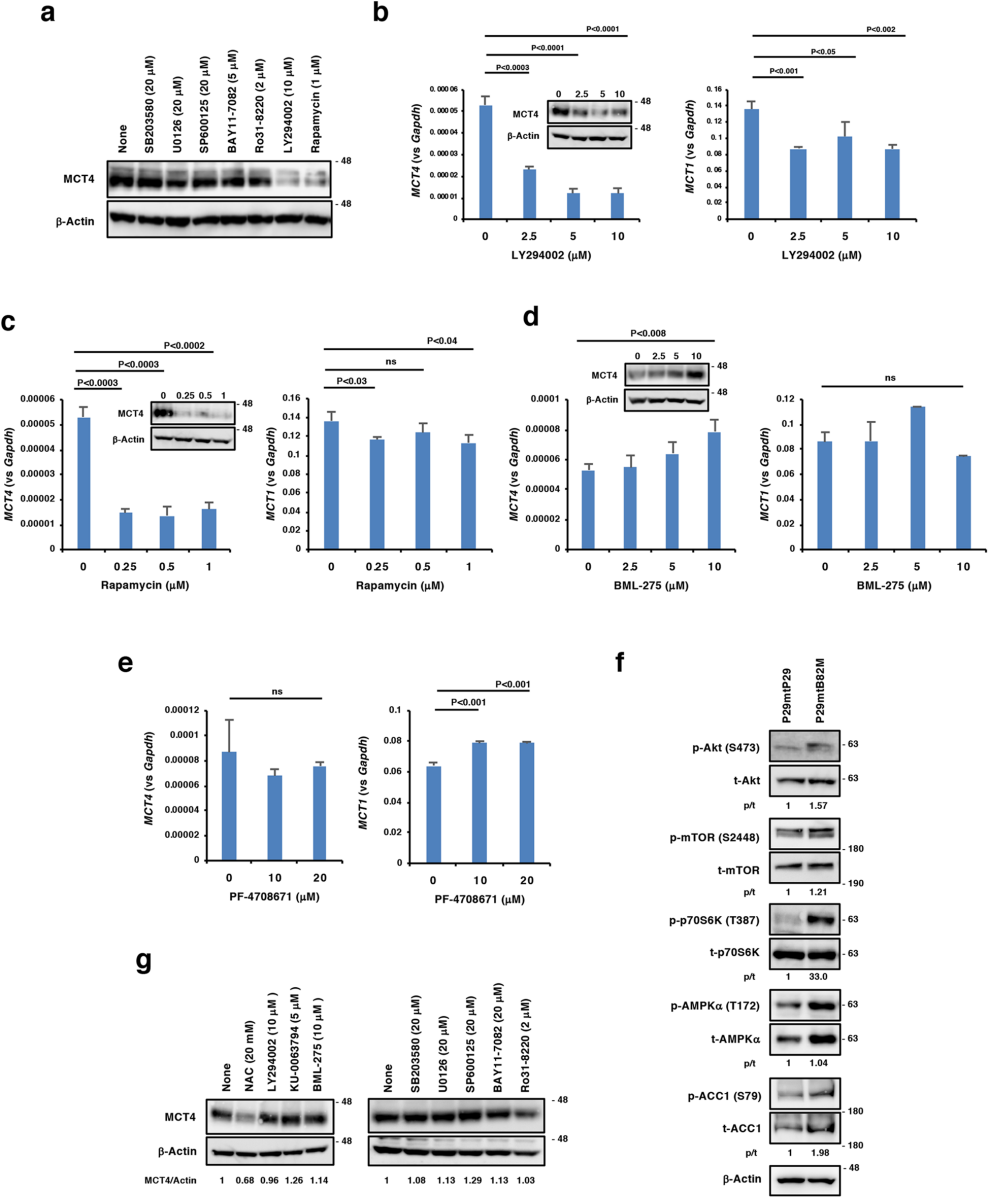
Analyses of signalling pathways involved in MCT4 expression in P29mtB82M and H358 cells by signalling pathway inhibitors. (**a**) Effects of various inhibitors on MCT4 expression in P29mtB82M cells. The cells were treated with the inhibitors at the indicated concentrations for 2 days. (**b**–**e**) Effect of LY294002, rapamycin, BML-275 and PF-4709671 on the expressions of MCT4 and MCT1. P29mtB82M cells were treated with LY294002 (**b**), rapamycin (**c**), BML-275 (**d**) or PF-4709671 (**e**) at the indicated concentrations for 2 days. The results of RT-qPCR and Western blotting are shown. (**f**) Comparison of phosphorylation levels of various signalling elements in P29mtP29 and P29mtB82M cells. Ratios of phosphorylated/total protein (p/t) are shown. (**g**) Effects of various inhibitors on MCT4 expression in H358 cells. Cells were treated with the inhibitors at the indicated concentrations for 2 days. For Western blotting, β-Actin was used as a loading control. Ratios of MCT4/β-actin are shown. Uncropped Western blot images are provided as Supplementary Fig. S12.

To further elucidate the biochemical mechanisms, we treated P29mtB82M cells with various signal transduction inhibitors, among which the phosphatidylinositol-3 kinase (PI3K) inhibitor LY294002 and the mechanistic target of rapamycin complex 1 (mTORC1) inhibitor rapamycin significantly suppressed MCT4 expression at both the mRNA and protein levels while only slightly affecting MCT1 expression (Fig. 7a–c). The p38 mitogen-activated protein kinase (MAPK) inhibitor SB203580, the mitogen-activated protein kinase kinase 1/2 (MEK1/2) inhibitor U0126, the c-Jun N-terminal kinase (JNK) inhibitor SP600125, the NF-κB inhibitor α (IκBα) inhibitor BAY11-7082 or the protein kinase C (PKC) inhibitor Ro31-8220 showed no effect on MCT4 expression (Fig. 7a). Interestingly, the AMP-activated protein kinase (AMPK) inhibitor BML-275 (Compound C) increased MCT4 expression but not MCT1 (Fig. 7d), while PF-4708671, a p70 S6 kinase (S6K) inhibitor, did not affect MCT4 expression even though MCT1 expression was slightly elevated (Fig. 7e). Altogether, these results suggest the involvement of Akt, mTOR and AMPK in regulating MCT4 expression. We then compared the activation status of these signalling molecules and downstream effectors between P29mtP29 and P29mtB82M cells, and found that Akt and its downstream mediators mTOR and p70 S6K were unhindered in the level of phosphorylation in P29mtB82M cells compared to P29mtP29 cells (Fig. 7f). AMPKα was constitutively overexpressed and phosphorylated at a higher level in P29mtB82M cells compared to P29mtP29 cells, a finding corroborated by the higher phosphorylation level in acetyl-CoA carboxylase 1 (ACC1), a key regulator in fatty acid biosynthesis directly downstream (Fig. 7f). Collectively, these results suggested the importance of PI3K-Akt-mTORC1 pathway activation, albeit p70 S6K is dispensable, for elevating MCT4 expression, alongside the action of AMPK activation in suppressing MCT4 expression in P29mtB82M cells.

Since *MCT4* transcription is said to be regulated by DNA methylation and histone acetylation^42,43^, we treated P29mtP29 cells with 5-aza-2’-deoxycytidine (5-aza-dC), a DNA methyltransferase 1 inhibitor, and trichostatin A (TSA), a histone deacetylase inhibitor, but found no induction of MCT4 expression in P29mtP29 cells (Supplementary Fig. S10). In addition, the treatment of P29mtP29 cells with the H3K9 lysine methyltransferase SUV39H1 inhibitor chaetocin also did not induce MCT4 expression (Supplementary Fig. S10); these results exclude the involvement of DNA methylation, histone acetylation and H3K9 methylation as the silencing mechanisms of MCT4 expression in P29mtP29 cells.

Unexpectedly, none of the inhibitors tested suppressed MCT4 expression in H375 cells (Fig. 7g), suggesting that signalling pathways regulating MCT4 expression in the cells are different from those in P29mtB82M cells.

### The MCT1/4 inhibitor syrosingopine kills lung cancer cells with pathogenic ND mutations

**Figure 8:**
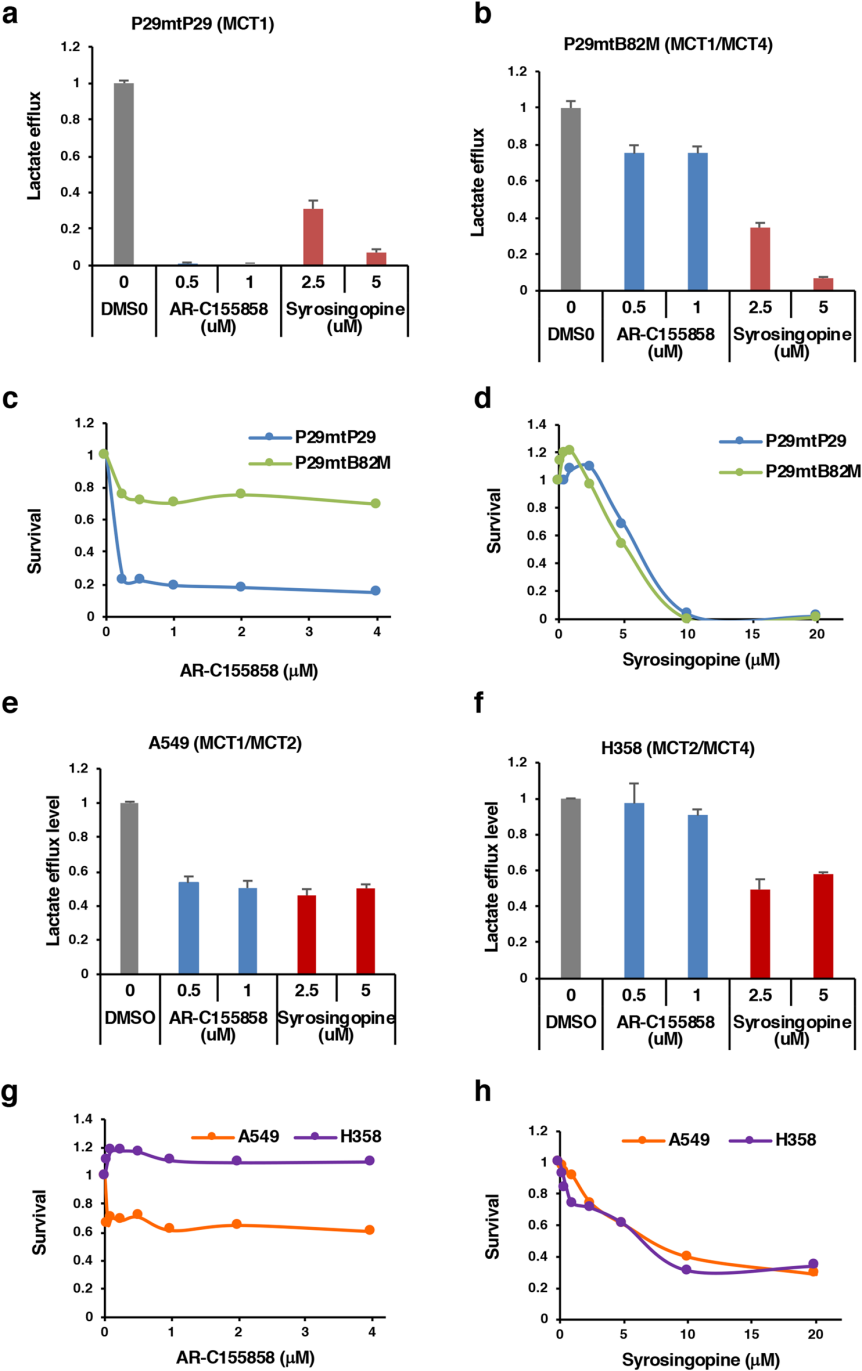
Inhibition of P29 cybrid cell and NSCLC cell growth by MCT inhibitors. (**a**, **b**) Effect of AR-C155858 and syrosingopine on lactate efflux in P29mtP29 and P29mtB82M cells. (**c**, **d**) Effect of AR-C155858 and syrosingopine on survival of P29mtP29 and P29mtB82M cells. (**e**, **f**) Effect of AR-C155858 and syrosingopine on lactate efflux in A549 and H358 cells. (**g**, **h**) Effect of AR-C155858 and syrosingopine on survival of A549 and H358 cells. Cell survival was determined by WST assay. Each point represents the mean of triplicate experimental results.

Finally, we examined whether cells with high levels of MCT4 expression that are associated with *ND* pathogenic mutations can be killed by MCT inhibitors. To this end, we employed the MCT1/2 inhibitor AR-C155858 and the MCT1/4 inhibitor syrosingopine. Treatment of P29mtP29 cells that expressed MCT1 with 1 μM AR-C155858 and 5 μM syrosingopine nearly completely inhibited lactate efflux (Fig. 7a). On the other hand, 1 μM AR-C155858 suppressed lactate efflux only slightly in P29mtB82M cells, probably due to additional MCT4 expression, while 5 μM syrosingopine suppressed the efflux (Fig. 8a,b). Accordingly, AR-C155858 markedly inhibited the survival of P29mtP29 cells but only slightly inhibited that of P29mtB82M cells (Fig. 8c). In contrast, syrosingopine killed both types of cells (Fig. 8d). The lactate efflux of A549 cells that expressed MCT1 and MCT2 was inhibited by approximately 50% by AR-C155858 and syrosingopine (Fig. 8e). Lactate efflux of H358 cells that expressed MCT2 and MCT4 was not inhibited by AR-C155858, which was probably due to MCT4 expression, but it was inhibited by 50% by syrosingopine (Fig. 8f). Coinciding with these results, AR-C155858 suppressed the survival of A549 cells but not H358 cells (Fig. 8g), while syrosingopine showed an approximately 70% reduction in survival in both cells (Fig. 8h). Thus, simultaneous inhibition of MCT1 and MCT4 was very effective in suppressing the growth of lung cancer cells with pathogenic *ND* mutations. Combination of AR-C155858 and syrosingopine did not enhance the death of H358 cells, compared to individual drugs (Supplementary Fig. S11).

## Discussion

Using a series of P29 cybrids in which we can specifically investigate the effect of mtDNA on the phenotypes and the pattern of gene expression, we tried to find a biomarker that correlated well with *ND* mutations and metastatic potential. We first examined the association between glucose uptake and the expression of glycolytic enzymes and metastasis but could not find any association. This seemed to coincide with our previous report that mtDNA mutation-enhanced glycolysis does not regulate metastasis^44^. Intriguingly, however, we found a clear association between pathogenic *ND6* mutations, MCT4 expression and metastatic potential. The association between MCT4 expression and metastatic potential was supported by siRNA-mediated MCT4 knockdown experiments in P29mtB82M cells in which invadopodia formation and Matrigel invasive potential were suppressed and the expression of invasion-related genes *MT1-MMP*, *MMP11*, *Plaur* and *TIMPs* was changed to suppress invasiveness. Involvement of MCT4 in EMT was excluded as siMCT4s did not consistently affect the expression of EMT-related genes. MCT1, MCT4 and CD147 have been shown to be involved in the invasive ability of different cancer cells, including breast and lung cancer cells^45,46^. Therefore, our results partly correspond to these previous reports. However, it should be stressed that only MCT4 was associated with pathogenic *ND6* mutations and distant metastatic potential, whereas neither MCT1 nor CD147 showed any association with such potential. At present, the precise mechanisms by which MCT4 affects the expression of invasion-related genes are unknown. Because MCTs have been implicated in CD147 proper membrane positioning^17^, MCT4 may enhance the activity of CD147 to induce MMPs^46^. Alternatively, because MCT4 is also reported to interact with CD44 in prostate cancer^47^, MCT4 may influence the role of CD44 in invasiveness. Further investigation using MCT4 knockdown P29mtB82M cells in vivo may provide a better light on the role of MCT4 in metastatic potential.

In the NSCLC cell lines examined, we predicted pathogenic *ND* mutations based on various criteria: the *ND5* G13708A mutation in H358 cells and the *ND4* G11453A mutation in RERF-Lc-Ad2 cells. Unfortunately, because cells with the same nucleus but with wild-type mtDNA are not available as a comparable control, we could not verify whether the two mutations are truly pathogenic by comparing complex I activity or oxygen consumption. However, the cells with each mutation produced a high level of mtROS, suggesting some disruption of complex I activity. Importantly, we exclusively detected MCT4 expression in the two cell lines. More importantly, strong MCT4 immunostaining was observed on the cell membrane of cancer cells in NSCLC tissue sections with predicted homoplasmic *ND* mutations. Although hypoxia contributed to the expression of MCT4 in cancer tissues, the staining was even throughout cancer tissue, suggesting that cancer cells outside the hypoxic areas also strongly expressed MCT4. In contrast, MCT4 staining of cancer cells without pathogenic *ND* mutations was uneven, localized in focal areas and relatively weak. Although strong MCT4 staining was observed in cancer cells in LgCa157 tissues that have no *ND* mutations, this might indicate the contribution of pathogenic mutations occurring in the nDNA-coded complex I subunit genes, which we should also consider because 45 subunits of complex I are encoded by both nDNA and mtDNA^48^.

A search of cancer survival databases showed a clear association between a higher expression of MCT4 and poorer outcomes in patients with NSCLC. It might be interesting to investigate the association between prognosis and frequency of pathogenic mutations in the *ND* genes and the nDNA-coded complex I subunit genes in patients with NSCLC as well as patients with other cancers.

The precise regulatory mechanisms of MCT4 expression in P29 cybrid cells and NSCLC cells remain unsolved. NAC inhibited MCT4 expression in both P29mtB82M cells and H358 cells. However, unexpectedly, the suppression was independent of ROS. Although we previously reported that ROS are responsible for the suppression of transcription of the antiapoptotic *MCL-1* gene^3^, MCT4 expression was not regulated by ROS, suggesting NAC-mediated actions other than ROS scavenging activity^49^ that may affect the transcription of the *MCT4* but not the *MCT1* gene expression. This indicates that both ROS-dependent and ROS-independent mechanisms are involved in the suppression of metastasis by NAC^3^. Another finding was that HIF-1 was excluded from the regulatory factors for MCT4 expression in normoxic P29mtB82M and H358 cells. Thus, although HIF-1 has been shown to play an important role in inducing MCT4 in some hypoxic cells, it does not necessarily contribute to its expression in cells with pathogenic *ND* mutations. Additionally, the PI3K-Akt-mTOR pathway and AMPK worked to enhance and suppress MCT4 expression in P29mtB82M cells, respectively. Accordingly, increased Akt, mTOR and AMPKα phosphorylation levels increased in situ; the pathogenic *ND6* 13891insC mutation in P29mtB82M cells likely caused complex I to dysfunction, leading to an increase in AMP which eventually activated AMPK, and subsequently suppressed MCT4 expression, while the PI3K-Akt-mTORC1 pathway worked in reverse to increase MCT4 expression significantly. This balance of AMP/ATP-AMPK and PI3K-Akt-mTORC1 pathways was presumably important for the regulation of MCT4 expression in P29mtB82M cells, although the activation of PI3K-Akt pathway in P29mtB82M cells still required further examination. In this scenario, PI3K activation by ROS-triggered oxidation of PTEN^50^ was excluded, while K-Ras and CD44, both of which were upregulated in P29mtB82M cells compared to P29mtP29 cells^4^, might be engaged in the activation^51^ instead. We advise that further studies on the mechanisms including signalling molecules downstream of mTORC1 and those of AMPK in P29mtB82 cells be performed, along with the identification of the silencing mechanisms of MCT4 expression in P29mtP29 cells. Results insofar would suggest the exclusion of DNA methylation, histone acetylation and H3K9 methylation; perhaps other epigenetics such as phosphorylation or sumoylation of transcriptional factors of the *MCT4* gene and/or miRNAs was mechanistically involved in the silencing mechanisms. Careful scrutiny of the RNA-seq data could provide additional clues for the regulatory puzzle in a future study. Unexpectedly, we found that PI3K, mTOR and AMPK did not contribute to MCT4 expression in H358 cells, indicating the possible difference in the regulatory pathways of MCT4 expression in different cells.

MCTs, especially MCT1 and MCT4, have attracted attention as therapeutic targets in cancer^29,32–36^. Small-molecule MCT1/2 inhibitors such as AR-C155858 and AZD3965 suppressed the proliferation of cancer cells expressing MCT1 but were ineffective against cancer cells that expressed both MCT1 and MCT4^52^. Notably, a recent study has demonstrated that the anti-hypertensive drug syrosingopine is a dual MCT1 and MCT4 inhibitor and that it elicited synthetic lethality when used with metformin, an inhibitor of complex I activity, to treat human cancer cells^30^. This implied that metastatic cells with pathogenic *ND* mutations may be killed by syrosingopine in the absence of metformin. As expected, we found that syrosingopine, but not AR-C155858, efficiently inhibited the survival of P29mtB82M and H358 cells. This suggests that dual inhibition of MCT1 and MCT4 by drugs such as syrosingopine represents a rational therapeutic approach to treat pathogenic *ND* mutation-mediated metastasis.

In conclusion, the present results suggested that pathogenic *ND* mutations are responsible for MCT4 induction in NSCLC cells and tissues. The involvement of the mutations in the nDNA-coded complex I subunit genes could also participate in the induction, which needs to be investigated. An important point is that, although the underlying mechanisms of its induction await to be resolved, MCT4 is suggested to be a biomarker and a therapeutic target of pathogenic *ND* mutation-harbouring metastatic tumours. Further studies to evaluate the feasibility of using MCT4 for such purposes are necessary in the future.

## Methods

### Reagents

Deferoxamine (DFO), CoCl_2_, 2-methoxyestradiol (2-ME), N-acetyl-L-cysteine (NAC), MitoTEMPO, U0126, 5-aza-2’-deoxycytidine (5-aza-dC), Y294002, SB203580, Ro31-8220, rapamycin, BML-275, trichostatin A (TSA) and 2’,7’-dichlorofluorescein diacetate (DCF-DA) were obtained from Sigma-Aldrich (St Louis, MO. USA), and MitoPQ, MitoSOX-Red and 2‐(N‐(7‐nitrobenz‐2‐oxa‐1,3‐diazol‐4‐yl)amino)‐2‐deoxyglucose (2‐NBD‐glucose) were from Abcam (Cambridge, UK), Thermo Fisher Scientific (Waltham, MA, USA) and Life Technologies (Carlsbad, CA, USA), respectively. AR-C155858 and SP600125 were supplied by Tocris Bioscience (Bristol, UK), and, syrosingopine was obtained from Extrasynthese SA (Lyon, France), respectively. TX-402 was a gift from Dr. H. Nagasawa, Gifu Pharmaceutical University. Rapamycin was purchased from LC Laboratories (Woburn, MA, USA); BAY11-7082 was from AdipoGen Life Sciences (San Diego, CA, USA); PF-4708671 and KU-0063794 were from Selleck Chemicals LLC (Houston, TX, USA), and chaetocin was from Enzo Life Science (Farmingdale, NY, USA).

### Cells and cell culture

The P29 cybrids, P29mtP29, P29mtΔ, P29mtCOI, P29mtA11 and P29mtB82M, were established by reintroducing wild-type mtDNA, mtDNA harbouring a 4696 bp deletion, T6589C in *COI*, G13997A in *ND6* or 13891insC in *ND6* into ρ^0^P29 derived from low-metastatic Lewis lung carcinoma P29 cells^3,4,12,44^ (Table 1), which were kindly provided by Dr Jun-Ichi Hayashi, Tsukuba University. *COI* T6589C and *ND6* G13997A and 13891insC are reported to be pathogenic mutations inducing significant complex IV and complex I defects, respectively^3,4,12,44^. Details of the derivation of human NSCLC cell lines, PC1, PC10 and A549, were described previously^53^. H358 and RERF-Lc-Ad2 cells were purchased from ATCC (Manassas, VA, USA). They were cultured in DMEM supplemented with 10% FBS, penicillin and streptomycin in a humidified atmosphere with 21% O_2_/5% CO_2_. Pyruvate (0.1 mg/ml) and uridine (50 mg/ml) were also added to the medium used in the culture of the P29 cybrids. These cells were free of mycoplasma contamination, as shown by tests performed with an e-Myco Mycoplasma PCR Detection Kit (Cosmo Bio Co Ltd., Tokyo, Japan).

### Ethics

All animal experiments were performed in compliance with the institutional guidelines for the care and use of animal research and the ARRIVE guidelines. The protocol was approved by the Committee on the Ethics of Animal Experiments of Chiba Cancer Center (Permission Number: 18-1). The use of the pathologic samples of NSCLC was approved and reviewed by the Ethics Committee of Chiba Cancer Center (Approval no. 20-14 and no. 21-1). All experiments were performed in accordance with relevant guidelines and regulations.

### Tumorigenicity and metastatic potential

Cybrid cells (2 × 10^5^ cells) were implanted subcutaneously into 6-week-old male C57BL/6 mice (CLEA Japan, Osaka, Japan). All mice were housed in a barrier facility under specific pathogen-free conditions at a controlled temperature of 23 ± 2 °C with 55 ± 10% humidity and 12 h light/12 h dark light cycles. The mice were maintained on a sterilized standard diet and water. After tumour transplantation, the mice were observed once a day to check their health. The tumour take rate was determined on Day 17. The mice were sacrificed on day 22, and the lungs were removed. After fixing in Bouin’s solution, the number of parietal metastatic foci was counted. Mice were euthanized by CO_2_ inhalation at the end of a study.

### Lactate efflux analysis

Cells were seeded at a density of 10,000 cells/100 μl/well in 0.5% FBS/DMEM in 96-well flat plates and then were incubated for 24 h. The amount of lactate in the conditioned medium was determined by a Lactate Assay Kit-WST (Dojindo Laboratories, Kumamoto, Japan).

### Glucose uptake analysis

Cybrid cells were incubated in serum‐free medium for 6 h before being washed once with PBS; then the cells were incubated with 100 μM 2‐NBD‐glucose for 1 h at 37 °C. Thereafter, the cells were detached by trypsinization, centrifuged at 300 g for 5 min and washed once with Dulbecco’s phosphate-buffered saline (DPBS). Cell pellets were resuspended in DPBS, and 2‐NBD‐glucose fluorescence was determined using a FACSCalibur flow cytometer (BD Biosciences, San Jose, CA, USA).

### Free fatty acid uptake analysis

TF2-C12 fatty acid uptake by the cybrids was determined with a Free Fatty Acid Uptake Assay Kit (fluorometric) (Abcam).

### ROS production

Mitochondrial ROS (mtROS) and cellular ROS generation were detected with the mitochondrial superoxide indicator MitoSOX-RED and DCF-DA, respectively. Cells were incubated with 5 μM MitoSOX-RED or 10 μM DCF-DA for 10 min at 37 °C in serum-free DMEM, washed twice with DPBS, and then immediately analysed with a FACSCalibur flow cytometer^3^.

### Western blotting

Cells were lysed in RIPA buffer containing cComplete Protease Inhibitor Cocktail (Merck, Kenilworth, NJ, USA) and PhosSTOP (Merck Millipore, Billerica, MA, USA). The lysates were centrifuged at 10,000 × g for 10 min at 4 °C, and the supernatants were used for immunoblot analysis according to our previously published method^3^. Proteins were separated by 10% SDS-PAGE under reducing conditions and then were transferred to Immobilon-P transfer membranes (Merck Millipore). The membranes were blocked with BLOCK ACE (DS Pharma Biomedical Co., Ltd) or 5% bovine serum albumin in TBS-T. The primary antibodies used were rabbit polyclonal anti-MCT1 (Santa Cruz Biotechnology), rabbit polyclonal anti-MCT4 (Santa Cruz Biotechnology), rabbit polyclonal anti-CD147 (Gene Tex, Irvine, CA, USA), mouse monoclonal anti-human HIF-1α (BD Biosciences, San Jose, CA, USA), rabbit polyclonal anti-HIF-1α (Gene Tex), rabbit polyclonal anti-Akt (Cell Signaling Technology, Danvers, MA, USA), rabbit polyclonal anti-Phospho-Akt (Ser473) (CST), rabbit monoclonal anti-mTOR, rabbit monoclonal anti-Phospho-mTOR (Ser2448) (CST), rabbit polyclonal anti-p70 S6 Kinase, rabbit polyclonal anti-Phospho-p70 S6 Kinase (Thr389), rabbit monoclonal anti-AMPKα, rabbit monoclonal anti-Phospho-AMPKα (Thr172) (CST), rabbit polyclonal anti-Acetyl-CoA Carboxylase 1 (ACC1), rabbit polyclonal anti-Phospho-Acetyl-CoA Carboxylase (Ser79) and mouse monoclonal anti-β-actin (Santa Cruz Biotechnology). All primary antibodies were used at 1:1000 dilutions. After primary antibody incubation, membranes were washed extensively with TBS-T and then incubated with the appropriate HRP-conjugated secondary antibody (1:3000 dilution). ECL Plus Western Blotting Detection Reagent (Amersham Biosciences, Piscataway, NJ) was used for immunodetection^3^. The density of the bands was analysed using NIH ImageJ 1.53 software.

### Immunocytochemistry

P29mtP29 and P29mtB82M cells were fixed with 4% formaldehyde and 5% sucrose in DPBS for 20 min, permeabilized with 0.5% Triton X-100 in DPBS for 5 min, and then blocked with 3% BSA/0.1% glycine in DPBS for 1 h according to our previously published method^3^. The cells were immunostained with a rabbit polyclonal anti-MCT4 antibody (1:200 dilutions) for 1 h, washed with DPBS, and then incubated with an Alexa Fluor 488-conjugated goat anti-rabbit antibody (Invitrogen, 1:300) for 1 h. The nuclei were counterstained with 1 μg/ml 4,6-diamidino-2-phenylindole (DAPI). The slides were observed under a confocal laser scanning microscope (Leica Microsystems, Wetzlar, Germany).

### Immunohistochemistry

Immunohistochemistry was performed according to our previously published method^3^. Briefly, archived paraffin-embedded primary and brain metastasis tissue samples from patients with NSCLC were cut into 4-μm sections. After deparaffinization and heat-induced antigen retrieval in REAL Target Retrieval Solution (DAKO), the tissues were treated with 0.3% hydrogen peroxide in methanol. MCT4 was detected with a mouse monoclonal anti-MCT4 antibody (SC-376140, 1:200 dilution, Santa Cruz Biotechnology, Dalla, TX, USA) and a VECTASTAIN Elite ABC HRP kit (Vector Laboratories, Burlingame, CA, USA). Nuclei were stained with haematoxylin. To semiquantitatively analyse the MCT4 immunostaining results, sections were given one of five IHC scores according to the percentage of MCT4-positive cells within the total cancerous cells: IHC score -, 0 positive; IHC score + , 1–10% positive; IHC score +  + , 11–50% positive; IHC score +  +  + , 51–90% positive; and IHC score +  +  +  + , > 90% positive.

### WST assay

Cells were seeded in the absence of drugs at a density of 10,000 cells/well in 96-well flat plates, and then cells were incubated overnight. The medium was then replaced with fresh medium containing AR-C155858 or syrosingopine, and then cells were incubated for 48 h. Cell viability was evaluated with a Cell Counting Kit-8 (Dojindo Laboratories).

### siRNA-mediated knockdown of MCT4

For the transient knockdown of MCT4, P29mtB82M cells were transfected with 20 nM MCT4 siRNA #1 (Slc16a3 Silencer Predesigned siRNA, ID:174957) and MCT4 siRNA #2 (ID: 174958) (Ambion, Thermo Fisher Scientific) using Lipofectamine RNAiMAX reagent (Thermo Fisher Scientific) according to the manufacturer’s protocol. Silencer Negative Control #1 siRNA (Ambion, Thermo Fisher Scientific) was used as a control. Two days after transfection, the cells were subjected to Western blot assays.

### Invadopodia assay

The ECM-degrading activity of P29mtB82M cells and cells transfected with MCT4 siRNA was assessed with a QCM Gelatin Invadopodia Assay (Green) kit (Merck Millipore, Burlington, MA, USA) according to the manufacturer’s protocol. The cells were seeded onto fluorescein-labelled gelatine and then were cultured for 24 h. After fixation with 4% formaldehyde in DPBS, the cells were stained with TRITC-phalloidin and DAPI. Fluorescent images were analysed by NIH ImageJ software, and the degradation area (pixel value) was normalized to the number of cells per field.

### Matrigel invasion assay

Cell invasion assays were performed with Corning BioCoat Matrigel Invasion Chambers (Corning, New York, USA). P29mtB82M cells and cells transfected with MCT4 siRNA were plated at a density of 1 × 10^5^ cells/well. The assay was performed according to the manufacturer’s instructions (Cell Biolabs, Inc., San Diego, CA, USA). Cells that had invaded the Matrigel were fixed and stained with 0.5% crystal violet, and the percentage of pores with stained cells was calculated.

### Sequencing of the ND gene

Sequencing of the *ND* genes was performed as previously described^4^. Briefly, the total DNA of NSCLC cells was isolated with QIAamp DNA Mini Kits (Qiagen, Hilden, Germany). The *ND* genes were amplified by PCR of the extracted DNA. The PCR conditions were as follows: 94 °C for 1 min followed by 30 cycles of amplification at 94 °C for 30 s, 53 °C for 30 s and 72 °C for 1 min, and there was a final extension at 72 °C for 1 min. PCR products were purified with a QIAquick PCR Purification Kit (Qiagen) and then were subjected to direct sequencing. The PCR primers are listed in Supplementary Table S3.

### RNA isolation and real-time quantitative PCR analysis

Total RNA was isolated using an RNeasy Plus Mini Kit (QIAGEN, Hilden, Germany) according to the manufacturer’s protocol. cDNA was synthesized using total RNA and ReverTra Ace qPCR RT Kit (TOYOBO, Osaka, Japan). Quantitative PCR was performed according to the protocol and consisted of an initial denaturation step at 95 °C for 1 min and 40 cycles of denaturation (95 °C for 15 s) and extension (60 °C for 1 min) as described previously^4^. The mRNA expression level was normalized to that of 18S ribosomal RNA (*Rn18s* for mouse, *RPS18* for human) or glyceraldehyde-3-phosphate dehydrogenase RNA (*Gapdh*). The primers used are summarized in Supplementary Table S3.

### RNA sequencing

Total RNA was extracted from P29mtP29 and P29mtB82M cells using an RNeasy Plus Mini Kit (QIAGEN). The quality and quantity of extracted total RNA were assessed using an Agilent 2100 BioAnalyzer and an RNA 6000 Nano Kit (Agilent Technologies, Santa Clara, CA, USA). Based on the quantification, 5 μg of total RNA with an RNA integrity number (RIN) value ≥ 7 was used for further steps. After depletion of ribosomal RNA with a RiboMinus Eukaryote System v2 (Thermo Fisher Scientific), the ribo-depleted RNA was subjected to preparation of barcoded libraries with the Ion Total RNA-Seq kit v2.0 (Thermo Fisher Scientific). The library was quantified using a High Sensitivity DNA Kit (Agilent Technologies) and was diluted to a final concentration of 8 pM. Following emulsion PCR using a One Touch system (Thermo Fisher Scientific) and an Ion PI Hi-Q OT2 200 Kit (Thermo Fisher Scientific), the prepared library was sequenced on P1v2 chips using an Ion Proton sequencer (Thermo Fisher Scientific).

### Sequencing data analysis

Reading were aligned using STAR^54^ to the mm10 reference mouse genome assembly, RNA-seq reads were processed into counts per million (CPM) and annotated by Subread^55^. For the purpose of differential expression profiling, we defined fold-changes, calculated using limma^56^ and edge R^57^, and defined as the difference in log_2_CPM for a given transcript between P29mtB82M and P29mtP29 such that a log FC > 0 indicated a higher CPM in P29mtB82M than was observed for the same transcript in P29mtP29. The results from individual transcripts were arithmetically averaged, and data were combined based on gene symbols using BioMart^58^ by querying Entrez GeneID and external gene names for subsequent pathway analysis. To determine the relative extent of differences in the magnitude of gene expression, the root-square deviation (RSD), or the square root of the square of the difference between P29mtB82M and P29mtP29, was adopted as the metric for comparison. Gene set pathway enrichment analysis was performed using the differentially expressed genes as input for all pathways under the mouse ("mmu") category in the Kyoto Encyclopedia of Genes and Genomes (KEGG)^59^, and statistical significance was evaluated for all mouse genes catalogued in KEGG using Fisher’s exact test and the predefined significance level of *p* < 0.01, when comparisons were made against the list of all genes that exist in a given mouse KEGG pathway. Mean expression changes in KEGG pathways were calculated from the mean logFC for matching symbols within a particular KEGG pathway. Part of the analysis and data processing involving the use of KEGG pathway was performed with custom scripts that were incorporated into the R package pipoft^60^. Raw sequencing reads were deposited to NCBI’s Short Read Archive (SRA) under the BioProject accession number PRJNA644940.

### Statistics

Statistical significance was tested using two-tailed Student’s t-test and one-way ANOVA. Statistical significance in the metastatic assay was tested by the Mann–Whitney U test. Unless otherwise specified, statistical significance was established at a predefined level of *p* < 0.05.

## Supplementary Information

Supplementary Information.

## Data Availability

The data generated or analysed in this study are included in this published article and its Supplementary Information files.

## References

[1] Lenaz G, Baracca A, Carelli V, D'Aurelio M, Sgarbi G, Solaini G (2004). Bioenergetics of mitochondrial diseases associated with mtDNA mutations. Biochim. Biophys. Acta.

[2] Gammage PA, Frezza C (2019). Mitochondrial DNA: the overlooked oncogenome?. BMC Biol..

[3] Ishikawa K (2008). ROS-generating mitochondrial DNA mutations can regulate tumour cell metastasis. Science.

[4] Koshikawa N, Akimoto M, Hayashi JI, Nagase H, Takenaga K (2017). Association of predicted pathogenic mutations in mitochondrial ND genes with distant metastasis in NSCLC and colon cancer. Sci. Rep..

[5] Hashizume O (2012). Specific mitochondrial DNA mutation in mice regulates diabetes and lymphoma development. Proc. Natl. Acad. Sci. USA.

[6] Marco-Brualla J (2019). Mutations in the ND2 subunit of mitochondrial complex I are sufficient to confer increased tumourigenic and metastatic potential to cancer cells. Cancers (Basel).

[7] Kulawiec M, Owens KM, Singh KK (2009). mtDNA G10398A variant in African-American women with breast cancer provides resistance to apoptosis and promotes metastasis in mice. J. Hum. Genet..

[8] Dasgupta S (2012). Mitochondrial DNA mutations in respiratory complex-I in never-smoker lung cancer patients contribute to lung cancer progression and associated with EGFR gene mutation. J. Cell Physiol..

[9] Santidrian AF (2013). Mitochondrial complex I activity and NAD+/NADH balance regulate breast cancer progression. J. Clin. Invest..

[10] Li LD (2015). Down-regulation of NDUFB9 promotes breast cancer cell proliferation, metastasis by mediating mitochondrial metabolism. PLoS ONE.

[11] Yuan Y (2015). Nonsense and missense mutation of mitochondrial ND6 gene promotes cell migration and invasion in human lung adenocarcinoma. BMC Cancer.

[12] Imanishi H (2011). Mitochondrial DNA mutations regulate metastasis of human breast cancer cells. PLoS ONE.

[13] Halestrap AP (2012). The monocarboxylate transporter family–Structure and functional characterization. IUBMB Life.

[14] Jones RS, Morris ME (2016). Monocarboxylate transporters: therapeutic targets and prognostic factors in disease. Clin. Pharmacol. Ther..

[15] Felmlee MA, Jones RS, Rodriguez-Cruz V, Follman KE, Morris ME (2020). Monocarboxylate transporters (SLC16): Function, regulation, and role in health and disease. Pharmacol. Rev..

[16] Ullah MS, Davies AJ, Halestrap AP (2006). The plasma membrane lactate transporter MCT4, but not MCT1, is up-regulated by hypoxia through a HIF-1alpha-dependent mechanism. J. Biol. Chem..

[17] Gallagher SM, Castorino JJ, Wang D, Philp NJ (2007). Monocarboxylate transporter 4 regulates maturation and trafficking of CD147 to the plasma membrane in the metastatic breast cancer cell line MDA-MB-231. Cancer Res..

[18] Koppenol WH, Bounds PL, Dang CV (2011). Otto Warburg's contributions to current concepts of cancer metabolism. Nat. Rev. Cancer.

[19] Pereira-Nunes A, Afonso J, Granja S, Baltazar F (2020). Lactate and lactate transporters as key players in the maintenance of the Warburg effect. Adv. Exp. Med. Biol..

[20] Zong WX, Rabinowitz JD, White E (2016). Mitochondria and cancer. Mol. Cell.

[21] Semenza GL (2013). HIF-1 mediates metabolic responses to intratumoural hypoxia and oncogenic mutations. J. Clin. Invest..

[22] Granja S, Marchiq I, Le Floch R, Moura CS, Baltazar F, Pouysségur J (2015). Disruption of BASIGIN decreases lactic acid export and sensitizes non-small cell lung cancer to biguanides independently of the LKB1 status. Oncotarget.

[23] Baek G (2014). MCT4 defines a glycolytic subtype of pancreatic cancer with poor prognosis and unique metabolic dependencies. Cell Rep..

[24] Chen HL (2018). Aberrant MCT4 and GLUT1 expression is correlated with early recurrence and poor prognosis of hepatocellular carcinoma after hepatectomy. Cancer Med..

[25] Cheng B, Chen X, Li Y, Huang X, Yu J (2018). Prognostic value of monocarboxylate transporter 4 in patients with esophageal squamous cell carcinoma. Oncol. Rep..

[26] Doyen J (2014). Expression of the hypoxia-inducible monocarboxylate transporter MCT4 is increased in triple negative breast cancer and correlates independently with clinical outcome. Biochem. Biophys. Res. Commun..

[27] Eilertsen M (2014). Monocarboxylate transporters 1–4 in NSCLC: MCT1 is an independent prognostic marker for survival. PLoS ONE.

[28] Gao HJ (2015). Monocarboxylate transporter 4 predicts poor prognosis in hepatocellular carcinoma and is associated with cell proliferation and migration. J. Cancer Res. Clin. Oncol..

[29] Nakayama Y (2012). Prognostic significance of monocarboxylate transporter 4 expression in patients with colorectal cancer. Exp. Ther. Med..

[30] Benjamin D (2018). Dual inhibition of the lactate transporters MCT1 and MCT4 is synthetic lethal with metformin due to NAD+ depletion in cancer cells. Cell Rep..

[31] Kong SC (2016). Monocarboxylate transporters MCT1 and MCT4 regulate migration and invasion of pancreatic ductal adenocarcinoma cells. Pancreas.

[32] Lamb R, Harrison H, Hulit J, Smith DL, Lisanti MP, Sotgia F (2014). Mitochondria as new therapeutic targets for eradicating cancer stem cells: quantitative proteomics and functional validation via MCT1/2 inhibition. Oncotarget.

[33] Morais-Santos F (2015). Targeting lactate transport suppresses in vivo breast tumour growth. Oncotarget.

[34] Todenhöfer T (2018). Selective inhibition of the lactate transporter MCT4 reduces growth of invasive bladder cancer. Mol. Cancer Ther..

[35] Kelsey R (2016). Prostate cancer: MCT4 is a novel target for prostate cancer. Nat. Rev. Urol..

[36] Choi SY (2016). The MCT4 gene: a novel, potential target for therapy of advanced prostate cancer. Clin. Cancer Res..

[37] Doherty JR, Cleveland JL (2013). Targeting lactate metabolism for cancer therapeutics. J. Clin. Invest..

[38] Xie L (2019). High KRT8 expression independently predicts poor prognosis for lung adenocarcinoma patients. Genes (Basel).

[39] Jones RS, Parker MD, Morris AME (2020). Monocarboxylate transporter 6-mediated interactions with prostaglandin F2α: in vitro and in vivo evidence utilizing a knockout mouse model. Pharmaceutics.

[40] Masoud GN, Li W (2015). HIF-1α pathway: role, regulation and intervention for cancer therapy. Acta Pharm. Sin. B.

[41] Akimoto M, Nagasawa H, Hori H, Uto Y, Honma Y, Takenaga K (2013). An inhibitor of HIF-α subunit expression suppresses hypoxia-induced dedifferentiation of human NSCLC into cancer stem cell-like cells. World J. Med. Genet..

[42] Fisel P (2013). DNA methylation of the SLC16A3 promoter regulates expression of the human lactate transporter MCT4 in renal cancer with consequences for clinical outcome. Clin. Cancer Res..

[43] Radoul M (2019). HDAC inhibition in glioblastoma monitored by hyperpolarized 13C MRSI. NMR Biomed..

[44] Ishikawa K (2008). Enhanced glycolysis induced by mtDNA mutations does not regulate metastasis. FEBS Lett..

[45] Izumi H (2011). Monocarboxylate transporters 1 and 4 are involved in the invasion activity of human lung cancer cells. Cancer Sci..

[46] Sun J, Hemler ME (2001). Regulation of MMP-1 and MMP-2 production through CD147/extracellular matrix metalloproteinase inducer interactions. Cancer Res..

[47] Pinheiro C, Reis RM, Ricardo S, Longatto-Filho A, Schmitt F, Baltazar F (2010). Expression of monocarboxylate transporters 1, 2, and 4 in human tumours and their association with CD147 and CD44. J. Biomed. Biotechnol..

[48] Formosa LE, Dibley MG, Stroud DA, Ryan MT (2018). Building a complex complex: assembly of mitochondrial respiratory chain complex I. Semin. Cell Dev. Biol..

[49] Aldini G (2018). N-Acetylcysteine as an antioxidant and disulphide breaking agent: the reasons why. Free Radic. Res..

[50] Kim JH (2018). Mitochondrial ROS-derived PTEN oxidation activates PI3K pathway for mTOR-induced myogenic autophagy. Cell Death Differ..

[51] Ouhtit A, Rizeq B, Saleh HA, Rahman MM, Zayed H (2018). Novel CD44-downstream signaling pathways mediating breast tumor invasion. Int. J. Biol. Sci..

[52] Polański R (2014). Activity of the monocarboxylate transporter 1 inhibitor AZD3965 in small cell lung cancer. Clin. Cancer Res..

[53] Akimoto M, Iizuka M, Kanematsu R, Yoshida M, Takenaga K (2015). Anticancer effect of ginger extract against pancreatic cancer cells mainly through reactive oxygen species-mediated autotic cell death. PLoS ONE.

[54] Dobin CA (2013). STAR: ultrafast universal RNA-seq aligner. Bioinformatics.

[55] Liao Y, Smyth GK, Shi W (2013). The Subread aligner: fast, accurate and scalable read mapping by seed-and-vote. Nucleic Acids Res..

[56] Ritchie ME, Phipson B, Wu D, Hu Y, Law CW, Shi W, Smyth GK (2015). limma powers differential expression analyses for RNA-sequencing and microarray studies. Nucleic Acids Res..

[57] Robinson MD, McCarthy DJ, Smyth GK (2010). edgeR: a Bioconductor package for differential expression analysis of digital gene expression data. Bioinformatics.

[58] Durinck S (2005). BioMart and Bioconductor: a powerful link between biological databases and microarray data analysis. Bioinformatics.

[59] Kanehisa M, Goto S (2000). KEGG: Kyoto encyclopedia of genes and genomes. Nucleic Acids Res..

[60] Lin J, Krishnamurthy S, Yoda H, Shinozaki Y, Watanabe T, Koshikawa N, Takatori A, Horton P, Nagase H (2019). Estimating genome-wide off-target effects for pyrrole-imidazole polyamide binding by a pathway-based expression profiling approach. PLoS ONE.

