# Supplementary material for: MCT4 is induced by metastasis-enhancing pathogenic mitochondrial NADH dehydrogenase gene mutations and can be a therapeutic target
Source: Sci Rep. 2021 Jun 25;11:13302. doi: 10.1038/s41598-021-92772-1 (PMC8233425; doi:10.1038/s41598-021-92772-1)
Supplement: Supplementary file 1 — Supplementary Information. [file 41598_2021_92772_MOESM1_ESM.pdf]

Supplementary information

MCT4 is induced by metastasis-enhancing pathogenic mitochondrial NADH dehydrogenase gene mutations and can be a therapeutic target

Keizo Takenaga, Nobuko Koshikawa, Miho Akimoto, Yasutoshi Tatsumi, Jason Lin,

Makiko Itami, Hiroki Nagase

Supplementary Table S1. SNVs found in *ND* genes in NSCLC cell lines

NSCLC cell line	ND1	ND2	ND3	ND4L	ND4	ND5	ND6
PC1 (squamous cell carcinoma)		C5178A (L237M)	A10398G/C10400T (T114A)				
PC10 (squamous cell carcinoma)		C5178A (L237M)	A10398G/C10400T (T114A)			G12503A (C56Y) (40% heteroplasmy)	T14180C (Y165C)
A549 (adenocarcinoma)		T4587C (F40L)				A13105G (I257V)	A14582G (V31A)
H358 (bronchioalveolar carcinoma)	T4216C (Y304H)		T10084C (I9T)			G13708A (A458T)	
RERF-Lc-Ad2 (adenocarcinoma)					G11453A (A232T)		T14447C (E76G)

Novel mutation

Pathogenic (predicted)

High Grantham value and associated with diseases

SNP

Not pathogenic (predicted)

Supplementary Table S2. Properties of nonsynonymous SNVs in *ND* genes in NSCLC cell lines.*

Gene	Nucleotide change	Amino acid change	Grantham value	MutPred Score	Evolutionary conservation	Conformational change (SWISS-MODEL)	Disease association	Haplogroup marker	Frequency in Japanese**
ND1	T4216C	Y304H	83	0.611	No	No	LHON/Insulin resistance/Schizophrenia/ Multiple sclerosis (population-dependent)	Haplogroup JT	2/672 (0.3%)
ND2	C5178A	L237M	15	0.26	No		No	Haplogroup D	261/672 (38.84%)
	T4587C***	F40L	22	0.580	No	No	No		—
ND3	T10084C	I9T	89	0.414	No	No	No		11/672 (1.64%)
ND4	G11453A	A232T	58	0.742	Yes	No	No		—
ND5	G12503A*** (40% heteroplasmy)	C56Y	194	0.399	No	No	—		—
	A13105G	I257V	29	0.35	No	No	No	Haplogroup L0/L1	7/672 (1.04%)
	G13708A	A458T	58	0.409	No	Yes	LHON / Increased MS risk / higher freq in PD-ADS (conflicting reports)	Haplogroup J	19/672 (2.83%)
ND6	A14582G	V31A	64	0.452	No	No	No		1/672 (0.15%)
	T14447C***	E76G	98	0.689	No	No	—		—
	T14180C	Y165C	194	0.614	No	No	No		8/672 (1.19%)

*See Ref. 4.

**mtSNP database (http://mtsnp.tmig.or.jp/mtsnp/search_home.html)

***Novel mutation

Pathogenic (predicted)

High Grantham value and associated with diseases

Not pathogenic (predicted)

Attention

Supplementary Table S3. PCR primers used for qPCR.

Target*	F/R**	Sequence (5'-3')	Accession No.	Application
mMCT1	F	ACGCCGGAGTCTTTGGATTT	NM_009196.4	qPCR
	R	GGCAGCATTCCACAATGGTC		
mMCT2	F	TGCACGTCAACACAAAGTGG	NM_011391.1	qPCR
	R	TCTTGCTTTGGGGGTTTCCTT		
mMCT3	F	CCAAGGCTGTTGGACACTCT	NM_020516.2	qPCR
	R	AGGCCTGTGAAGGGTGAATG		
mMCT4	F	GGCGGTAACAGGTGAAAGCA	NM_001038654.1	qPCR
	R	GCGTAGGAGAAACCCGTGAT		
mMCT5	F	GACCCATCCACAGCCAATCA	NM_146136.2	qPCR
	R	GCCTCTGGTTCAGTTGCAGA		
mMCT6	F	TATCCACGTAGGGGAAGGCA	NM_001080934	qPCR
	R	GCAGAGCACAGACTACGGTT		
mMCT7	F	CCACGAACTGACAGACTGCT	NM_001029842	qPCR
	R	TGACTTGGGCTGGATGTCAC		
mMCT13	F	GGCGCCACATGCTTTATCAG	NM_172371.3	qPCR
	R	TAAGAGCCCCTGCCTGATCT		
mMCT14	F	GGCTGCGTGCAAAAACCTTA	NM_027921.1	qPCR
	R	CGATATCTGGGTGGGGCTTC		
mCD147	F	TGAAGGGAATGCTCCAAACGA	NM_009768.2	qPCR
	R	GCCCATCAACAGAGAGCGAA		
mMT1-MMP	F	CCCAAGGCAGCAACTTCA	NM_008608.4	qPCR
	R	CCCTGGAGGTAGGTAGCCATA		
mMMP2	F	CCCGAGACCGCTATGTCCACT	NM_008610.3	qPCR
	R	GCCCCACTTCCGGTCATCATCGTA		
mMMP9	F	GCGCCACCACAGCCAACTATG	NM_013599.4	qPCR
	R	TGGATGCCGTCTATGTCGTCTTTA		
mTIMP1	F	ACTCGGACCTGGTCATAAGGGC	NM_001044384.1	qPCR
	R	TTCCGTGGCAGGCAAGCAAAGT		
mTIMP2	F	GGCAACCCCATCAAGAGGA	NM_011594.3	qPCR
	R	CCTTCTGCCTTTCCTGCAATTAG		
mMMP11	F	CACCGTCATCACCCTGTGAA	NM_008606.3	qPCR
	R	GGTTCCGGGCATTCAGTACA		
mPlaur	F	ACTACCGTGCTTCGGGAATG	NM_011113.4	qPCR
	R	GCCTGTTGCAGAGGTTTGTG		
mGlut1	F	GCGGGAGACGCATAGTTACA	NM_011400	qPCR
	R	TAGCCGAACTGCAGTGATCC		
mHK1	F	CCCATCGCTTTAGTGAGCCA	NM_001146100.1	qPCR
	R	CACCCCAAGGAAACACCACT		
mHK2	F	CCCTGTGAAGATGTTGCCCAC	NM_013820.3	qPCR
	R	TGCCCATGTACTCAAGGAAGT		
mPKF1	F	GCAGCCTACAATCTGCTCCA	NM_008826.4	qPCR
	R	GTCAGTGCCGCAGAAGTCAT		
mGAPDH	F	TGCACCACCAACTGCTTAG	NM_001289726.1	qPCR
	R	GGATGCAGGGATGATGTTC		
mPGK1	F	TTGTGCATTGTAGAGGGCGT	NM_008828	qPCR
	R	TGACGAAGCTAACCAGAGGC		
mPKM1	F	TCCAGTCACTCCACAGACCT	NM_001253883.1	qPCR
	R	CCACTTGGTGAGCACTCCTG		
mPKM2	F	ATTACCAGCGACCCCACAG	NM_011099.3	qPCR
	R	CCACTTGGTGAGCACTCCTG		
mLHDA	F	ACTGCAGGCTTCGATTACCC	NM_010699.2	qPCR
	R	ATGGACGTACACACTGGAGC		
mLHDB	F	GGTGAAGGGAATGTACGGCA	NM_008492.2	qPCR
	R	GAGCGACCTCATCGTCCTTC		
mCol6a3	F	CTGGCTCTCACCCAGAAAGG	NM_001243008.1	qPCR
	R	TCAGTTCGGACAGTTCCTGC		
mCol5a3	F	AGAAAGGCGATCAGGGGTTG	NM_016919.3	qPCR
	R	GGTGACCTAACGAGCCAACA		

mKrt8	F	CGGGGGATCCAACACTTTCA	NM_031170.2	qPCR
	R	CAGCTTCCCATCTCGGGTTT		
mGatm	F	CACTTGAGTAACTTGGCCGC	NM_025961.5	qPCR
	R	TTGCCTATGTCTTTGTCCCAT		
mRn18s	F	CTTAGAGGGACAAGTGGCG	NR_003278.3	qPCR
	R	ACGCTGAGCCAGTCAGTGTA		
hMCT1	F	AGTTCGGATGTCTGTGTGGC	NM_003051.3	qPCR
	R	AGCGAGGCTGCCTTATAACC		
hMCT2	F	AGAGGAGCAGAAATGCCACC	NM_001270623.2	qPCR
	R	ATACGGTGACAGCTTTGGGG		
hMCT3	F	CTTTGCAGGAGAAGGAGACTTG	NM_013356.2	qPCR
	R	CTGTAGCCGGCGTCGAA		
hMCT4	F	GAGTTTGGGATCGGCTACAG	NM_001206950.1	qPCR
	R	CGGTTCACGCACACACTG		
hMCT6	F	GTGCGAGCGGTACAAAGAAA	NM_004695.4	qPCR
	R	ATTCCAGACTCAAGCAGGGC		
hMCT7	F	AGCCAACTTGCGCTAGAGTC	NM_001174166.1	qPCR
	R	CCCTCCTTATAAGCCGCTCG		
hMCT13	F	CTTTGCAGGAGAAGGAGACTTG	NM_201566.2	qPCR
	R	CTGTAGCCGGCGTCGAA		
hMCT14	F	CTTTCAGCTCCACCAAACGC	NM_152527.5	qPCR
	R	GCCTATCATCCTGGAGACGC		
hCD147	F	CCGTAGAAGACCTTGGCTCC	NM_001728.3	qPCR
	R	TACTCTCCCCACTGGTCGTC		
mSNAI1	F	CTGCTTCGAGCCATAGAACTAAAG	NM_011427.3	qPCR
	R	GAGGGGAACTATTGCATAGTCTGT		
mSNAI2	F	TCCCATTAGTGACGAAGA	NM_011415.3	qPCR
	R	CCCAGGCTCACATATTCC		
mTwist1	F	GCAAGATCATCCCCACGCTG	NM_011658.2	qPCR
	R	GCAGGACCTGGTAGAGGAAG		
mTwist2	F	CAGTGACTTCTGTGCCCTCA	NM_007855.3	qPCR
	R	TGAGAGCCTTGGTCCAGTTT		
mZeb1	F	ACTGCTGGCAAGACAACGTG	NM_011546.3	qPCR
	R	ATGACGGCGGTGTCTTGTTG		
mZeb2	F	GAGCAGGTAACCGCAAGTTC	NM_015753.4	qPCR
	R	TCTTTCAGGTGGTGCTTGTACT		
mVim	F	CGGCTGCGAGAGAAATTGC	NM_011701.4	qPCR
	R	CCACTTTCCGTTCAAGGTCAAG		
mCdh1	F	GTCTCCTCATGGCTTTGC	NM_009864.3	qPCR
	R	CTTTAGATGCCGCTTCAC		
hRPS18	F	GAGGATGAGGTGGAACGTGT	NM_022551.3	qPCR
	R	TCTTCAGTCGCTCCAGGTCT		
hND1	F	AACAGGGTTTGTTAAGATGGC	MH8073364.1	Sequencing
	R	GGTTCGATTCTCATAGTCCTA		
hND1	F	CATCAAACTCAAACTACGCCCT	MH8073364.1	Sequencing
	R	TTCGGTTGGTCTCTGCTAGT		
hND2	F	ATGAGAATCGAACCCATCCC	MH8073364.1	Sequencing
	R	TAAGATTTTGCGTAGCTGGGT		
hND2	F	GACATCCGGCCTGCTTCTT	MH8073364.1	Sequencing
	R	GAGTGGGGTTTTGCAGTCC		
hND3-ND4L	F	CCATCTATTGATGAGGGTCTT	MH8073364.1	Sequencing
	R	ATAATTAGGCTGTGGGTGGTT		
hND4	F	CTAGTATATCGCTCACACCTCA	MH8073364.1	Sequencing
	R	GCTTCGACATGGGCTTTAGGGA		
hND4	F	CTACTCACTCTCACTGCCCAAG	MH8073364.1	Sequencing
	R	GGGGAATTAGGGAAGTCAGGGT		
hND5	F	CTCACAAGAACTGCTAACTCATGCC	MH8073364.1	Sequencing
	R	CTAGTAGTGGGGTGAGGCTTGGATTA		
hND5	F	CATCGGCTGAGAGGGCGTAGGAAT	MH8073364.1	Sequencing
	R	GAGAGTAATAGATAGGGCTCAGGCG		
hND5	F	CTCCGGGTCCATCATCCACAACCT	MH8073364.1	Sequencing

hND5	R	GATCCTATTGGTGCGGGGGCTTTG	MH8073364.1	Sequencing
hND6	F	CAATTTCACAGCACCAAATCTCCA	MH8073364.1	Sequencing
	R	TATTAGGGGGTTAGTTTTGCG		

*m: Mouse, h: Human

**F: Forward, R: Reverse

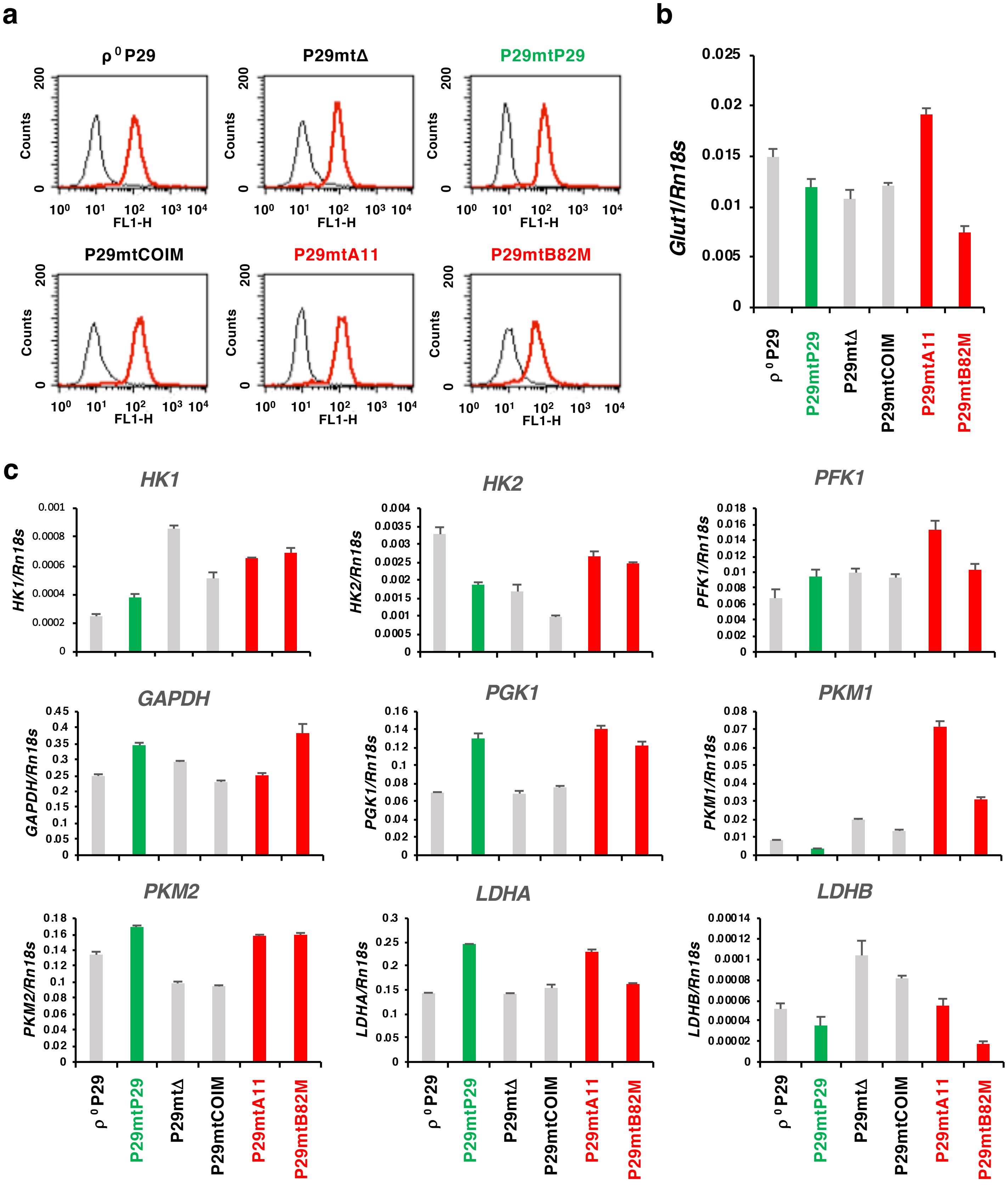

Supplementary Fig. S1. Glucose uptake and the expression of glucose transporter-1 and glycolytic enzymes in the cybrids. (a) 2-NBD-glucose uptake. (b) RT-qPCR analysis of the expression of *Glut1*. (c) RT-qPCR analysis of the expression of glycolytic enzyme genes.

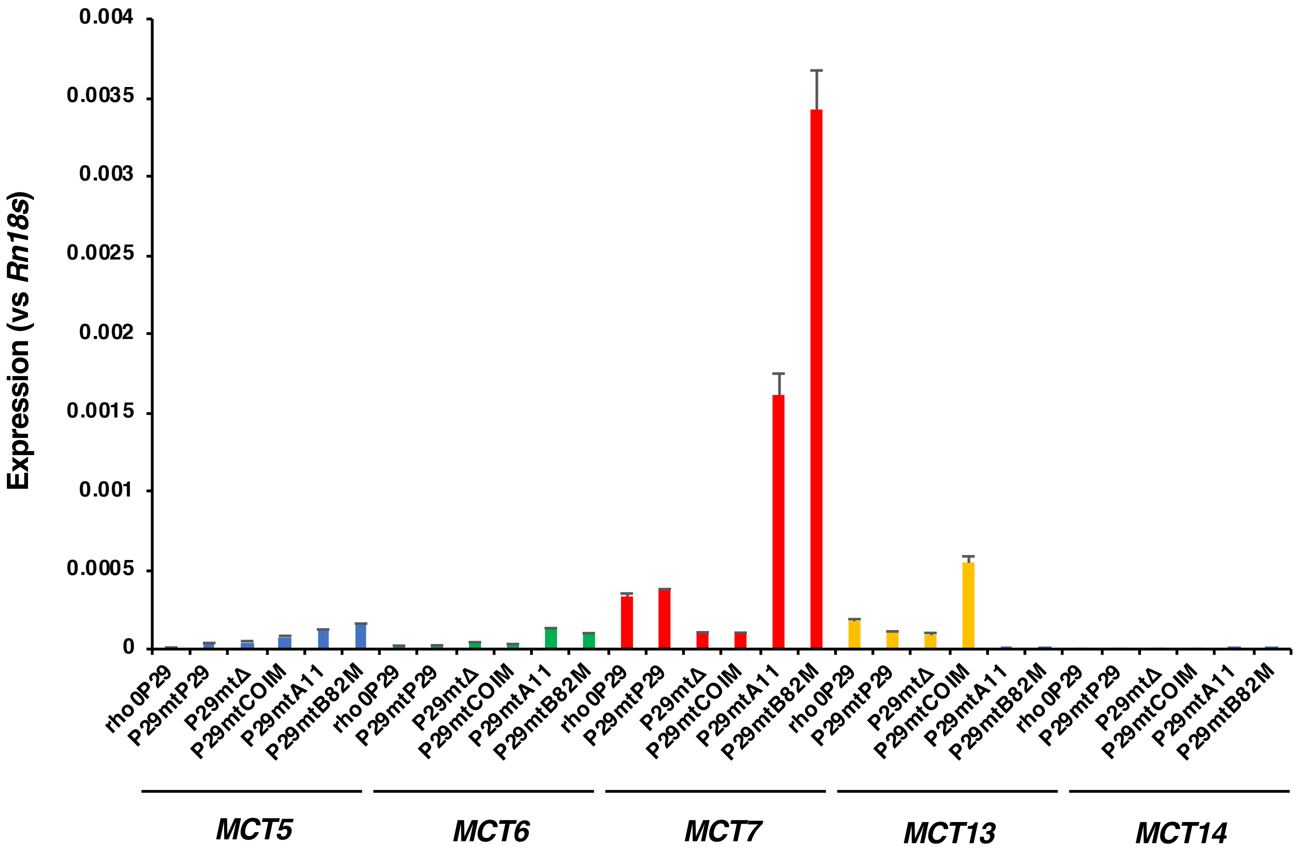

Supplementary Fig. S2. RT-qPCR analysis of the expression of MCTs in P29 cybrids.

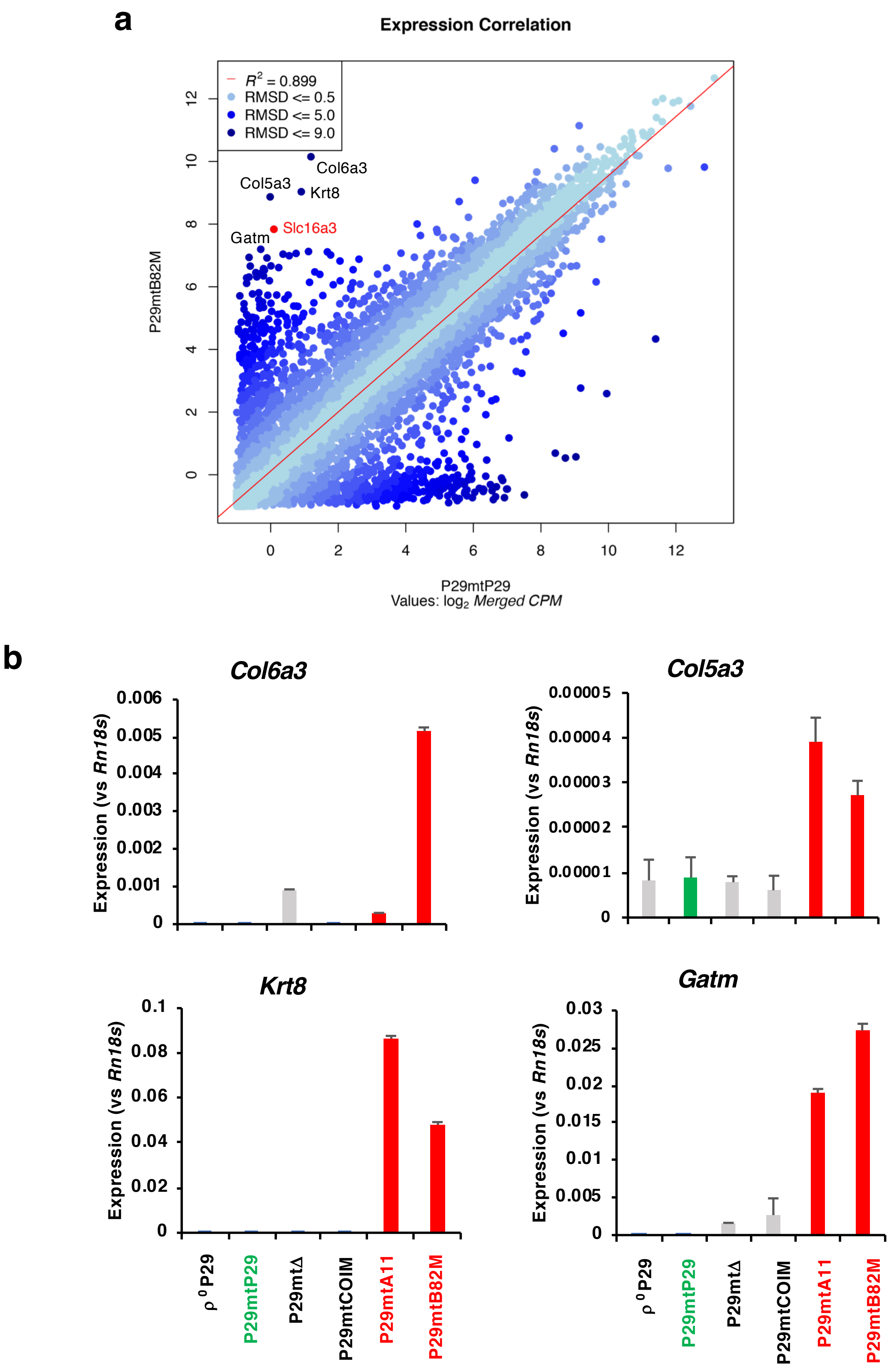

Supplementary Fig. S3. RNA-seq analysis and the expression of selected genes in P29 cybrids. (a) RNA-seq analysis of the differentially expressed genes in P29mtP29 and P29mtB82M cells. (b) RT-qPCR analysis of the expression of *Col6a3*, *Col5a3*, *Krt8* and *Gatm* in P29 cybrids.

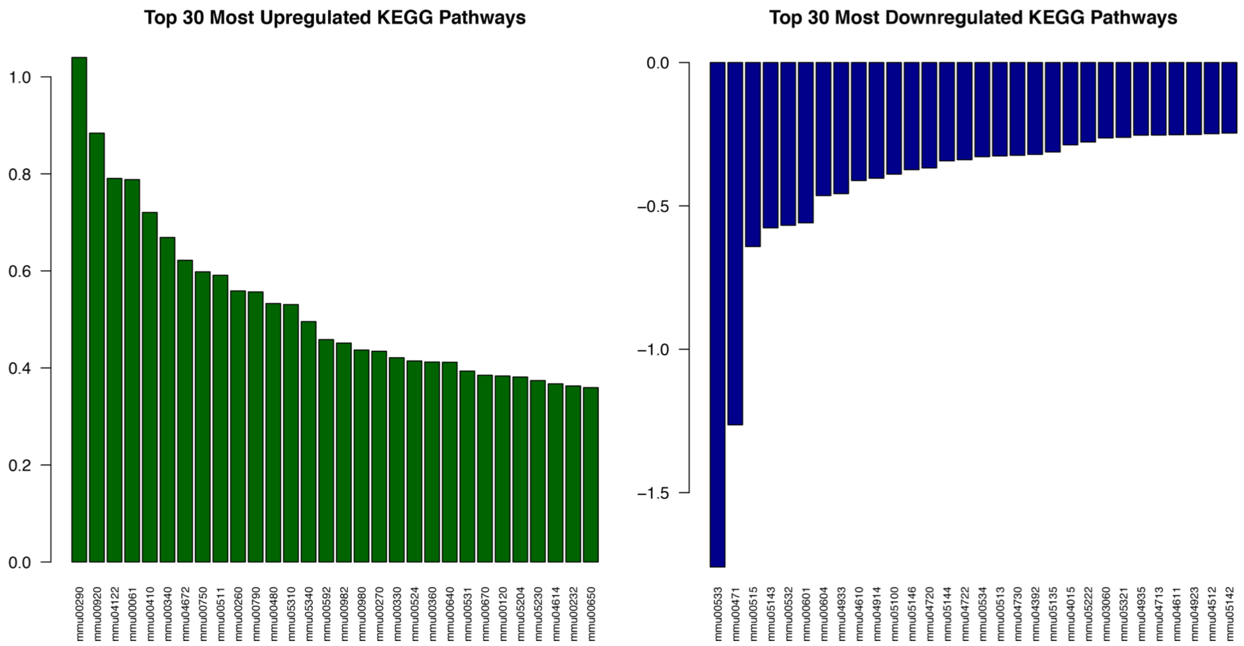

Top 30 Most Upregulated KEGG Pathways	
mmu00290	Valine, leucine and isoleucine biosynyhesis
mmu00920	Sulfur metabolism
mmu04122	Sulfur relay system
mmu00061	Fatty acid biosynthesis
mmu00410	beta-Alanine metabolism
mmu00340	Histidine metabolism
mmu04672	Intestinal immune network for IgA production
mmu00750	Vitamin B6 metabolism
mmu00511	Other glycan degradatiom
mmu00260	Glycine, serine and threonine metabolism
mmu00790	Folate biosynthesis
mmu00480	Glutathione metabolism
mmu05310	Asthma
mmu05340	Primary immunodeficiency
mmu00592	alpha-Linolenic acid metabolism
mmu00982	Drug metabolism - cytochrome P450
mmu00980	Metaolism of xrenobiotics by cytochrome P450
mmu00270	Cysteine and methionine metabolism
mmu00330	Arginine and proline metabolism
mmu00524	Neomycin, kanamycin and gentamicin biosynthesis
mmu00360	Phenylalanine metabolism
mmu00640	Propanoate metabolism
mmu00531	Glycosaminoglycan degradation
mmu00670	One carbon pool by folate
mmu00120	Primary bile acid biosynthesis
mmu05204	Chemical carcinigenesis
mmu05230	Central carbon metabolism in cancer
mmu04614	Renin-angiotensin system
mmu00232	Caffeine metabolism
mmu00650	Butanoate metabolism

Top 30 Most Downregulated KEGG Pathways	
mmu00533	Glycosaminoglycan biosynthesis - keratan sulfate
mmu00471	D-Glutamine and FD-glutamate metabolism
mmu00515	Mannose type O-glycan biosynthesis
mmu05143	African trypanosomiasis
mmu00532	Glycosaminoglycan biosynthesis - chondroitin sulfate / dermatan sulfate
mmu00601	Glycosphingolipid biosynthesis - lacto and neolacto series
mmu00604	Glycosphingolipid biosynthesis - ganglio series
mmu04933	AGE-RAGE signaling pathway in diabetic complications
mmu04610	Compliment and coagulation cascades
mmu04914	Progesterone-mediated oocyte maturation
mmu05100	Bacterial invasion of epithelial cells
mmu05146	Amoebiasis
mmu04720	Long-term potentiation
mmu05144	Malaria
mmu04722	Neurotropin signaling pathway
mmu00534	Glycosaminoglycan biosynthesis - heparan sulfate
mmu00513	Various types of N-glycan biosysnthesis
mmu04730	Lomg-term depression
mmu04392	Hippo signaling pathway - multiple species
mmu05135	Yersinia infection
mmu04015	Rap1 signaling pathway
mmu05222	Small cell lung cancer
mmu03060	Protein export
mmu05321	Inflammatory bowel disease (IBM)
mmu04935	Growth hormone synthesis, secretion and action
mmu04713	Circadian entrainment
mmu04611	Platelet activation
mmu04923	Regulation of lipolysis in adipocytes
mmu04512	ECM-receptor interaction
mmu05142	Chagas disease (American trypanosomiasis)

Supplementary Fig. S4. Top 30 most upregulated and downregulated KEGG pathways in P29mtB82M cells compared to P29mtP29 cells. In the left table, the pathways shown in red indicate those related to amino acid biosynthesis. In the right table, the pathways shown in blue indicate those related to glycosaminoglycan and glycosphingolipid biosynthesis.

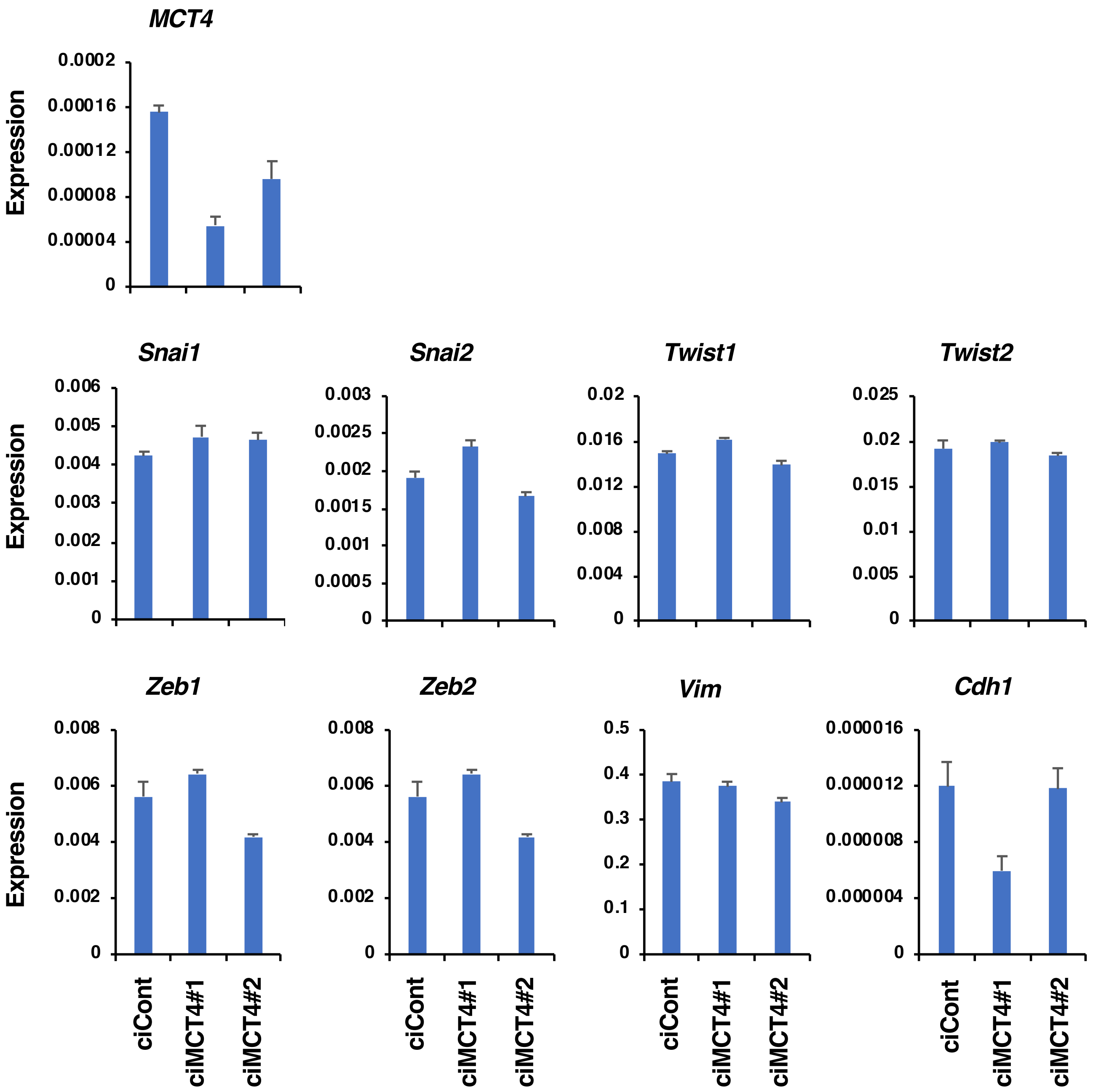

Supplementary Fig. S5. RT-qPCR analysis of the expression of MCT4 and EMT-related genes in P29mtB82M cells transfected with a control siRNA (siCont) or MCT4 siRNA (siMCT4 #1 or #2). Error bar: SD.

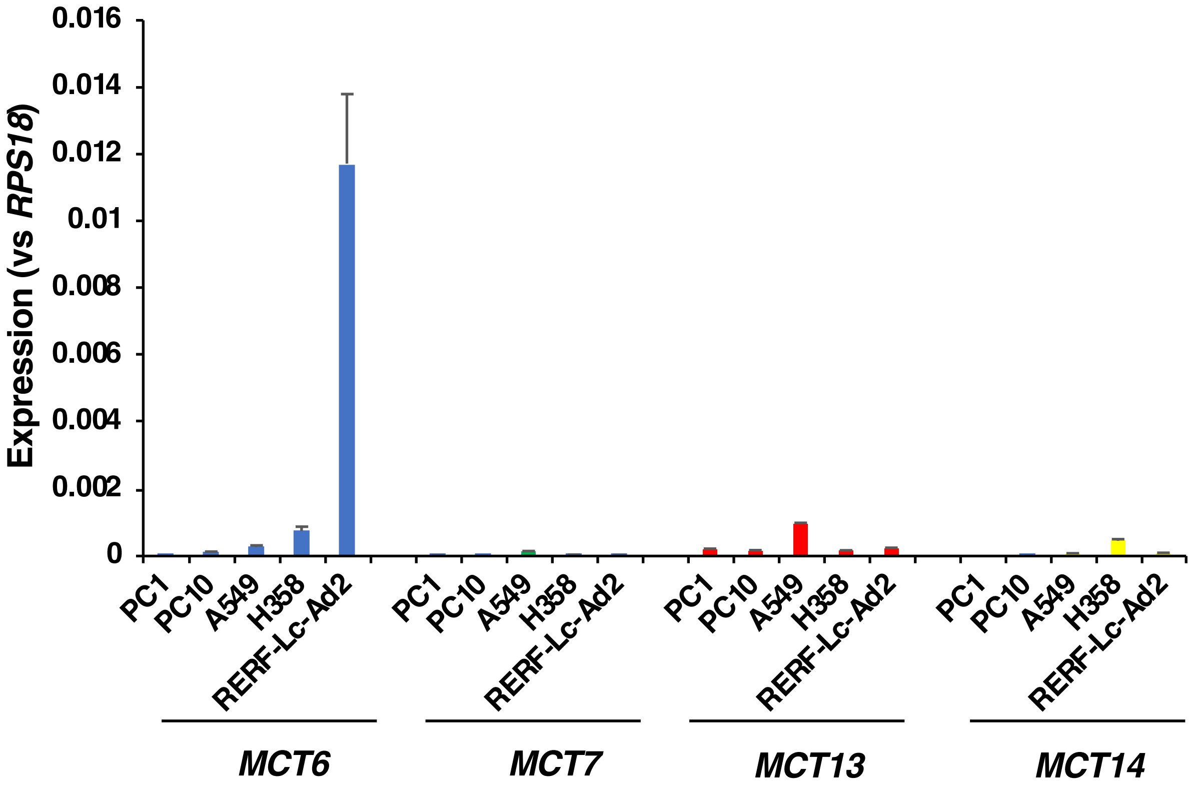

Supplementary Fig. S6. RT-qPCR analysis of the expression of *MCT6*, *MCT7*, *MCT13* and *MCT14* in NSCLC cell lines.

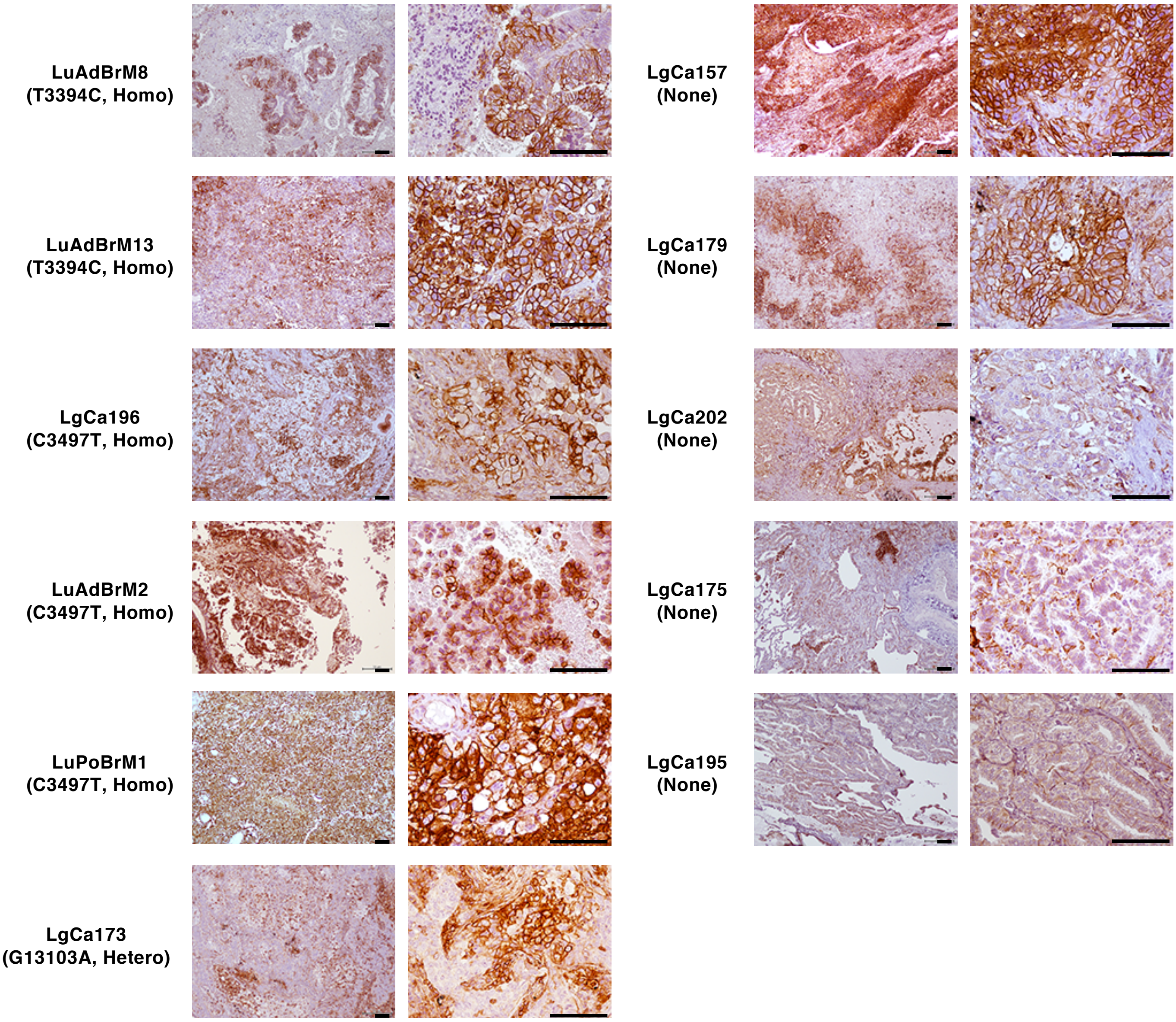

Supplementary Fig. S7. IHC analysis of MCT4 expression in NSCLC tissues. NSCLC tissues harbouring predicted pathogenic ND mutations such as homoplasmy (LuAdBrM8, LuAdBrM13, LgCa196, LuAdBrM2 and LuPoBrM1) or heteroplasmy (LgCa173) and NSCLC tissues having no ND pathogenic mutation (LgCa157, LgCa179, LgCa202, LgCa175 and LgCa195) were immunostained for MCT4 using a monoclonal MCT4 antibody. BrM and LgCa indicate brain metastasis and primary lung tumour, respectively. Bar: 100 µm.

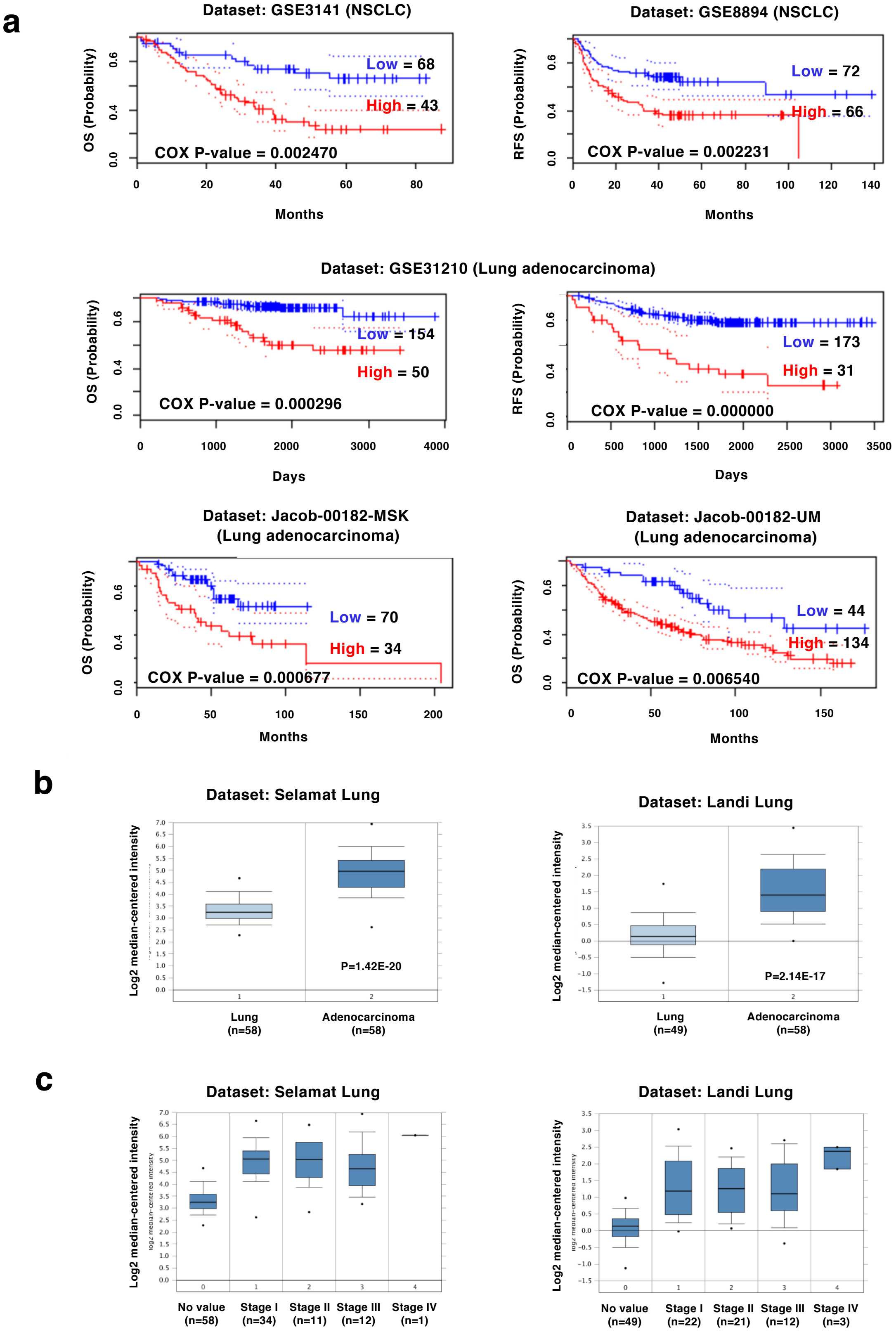

Supplementary Fig. S8. Correlation between MCT4 expression and prognosis in NSCLC patients. (a) Association of MCT4 expression and overall survival (OS) and relapse-free survival (RFS). Five datasets in PROGgeneV2 (http://watson.compbio.iupui.edu/chirayu/proggene/database/?url=proggene) are shown. (b) MCT4 expression in normal lung and lung adenocarcinomas. (c) MCT4 expression in cancer tissues from patients with different stages of lung adenocarcinomas. The datasets from the Oncomine database (https://www.oncomine.org/resource/login.html) are shown.

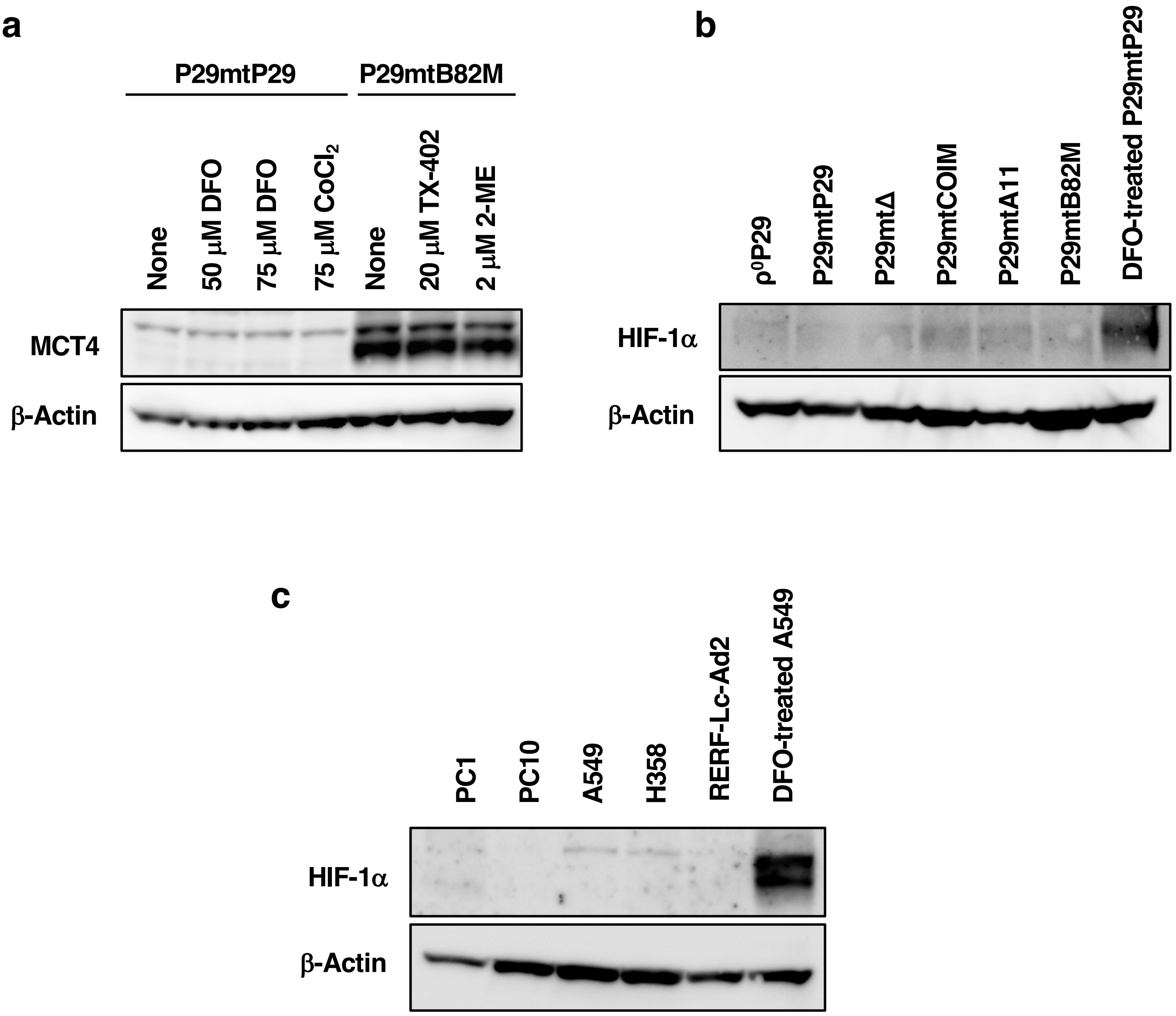

Supplementary Fig. S9. Western blot analyses of the relationship between HIF-1 and MCT4 expression. (a) Effect of HIF-1 activators and inhibitors in P29 cybrids. The cells were treated with the indicated drugs for 2 days. β-Actin was used as a loading control. (b) HIF-1α expression in the cybrids. Lysate from P29mtP29 cells treated with 250 μM DEO for 6 h was used as a control. β-Actin was used as a loading control. (c) Western blot analysis of HIF-1α expression in NSCLC cell lines. Lysate from A549 cells treated with 250 μM DFO for 4 h was used as a control. β-Actin was used as a loading control. Uncropped Western blot images are shown in Supplementary Fig. S12.

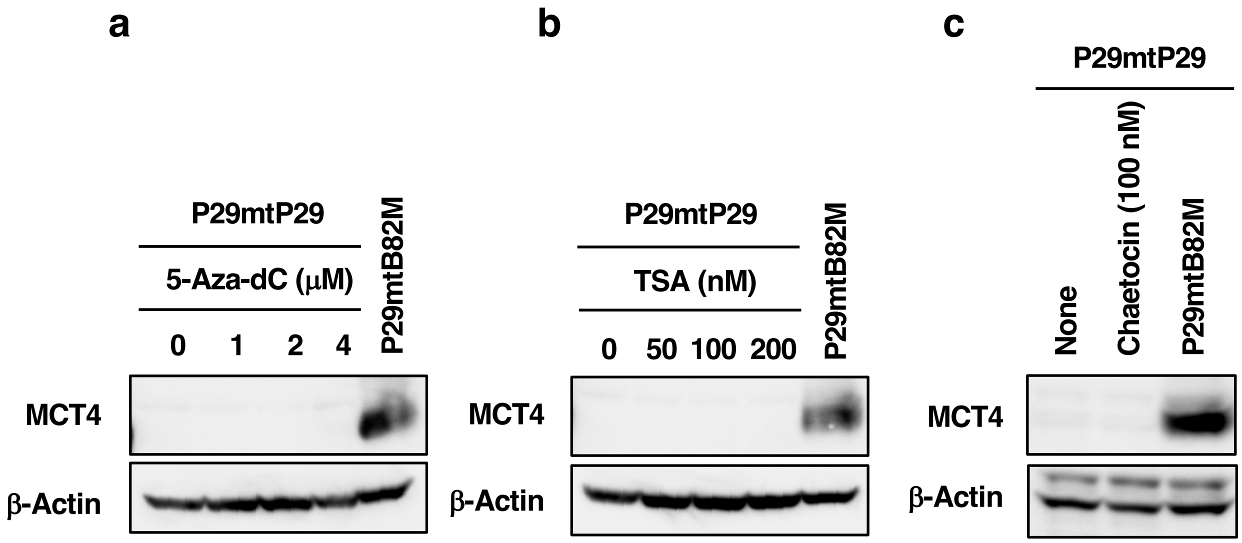

Supplementary Fig. S10. Western blot analyses of the effects of 5-aza-dC (a), TSA (b) and chaetocin (c) on MCT4 expression in P29mtP29 cells. The cells were treated with the drugs at the indicated concentrations for 2 days. β-Actin was used as a loading control. Uncropped Western blot images are shown in Supplementary Fig. S12.

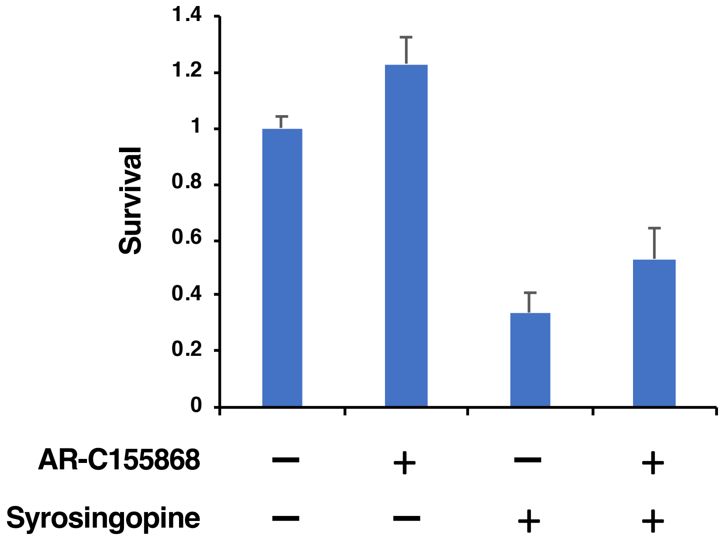

Supplementary Fig. S11. Combinatory effect of AR-C155868 and syrosingopine on the survival of H358 cells; cells were treated with 4 μM AR-C155868, 10 μM syrosingopine and their combination for 2 days. Error bar: SD.

Fig. 1d

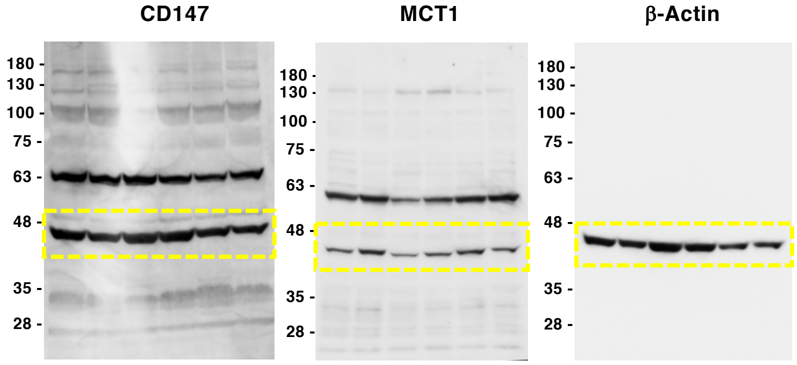

Fig. 1f

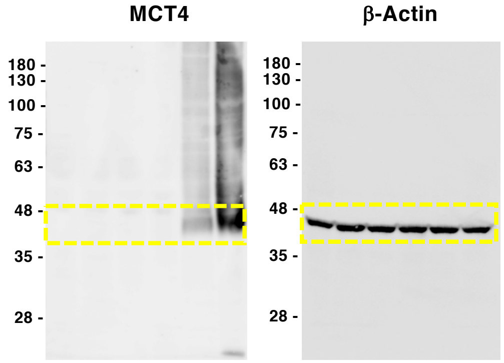

Fig. 2a

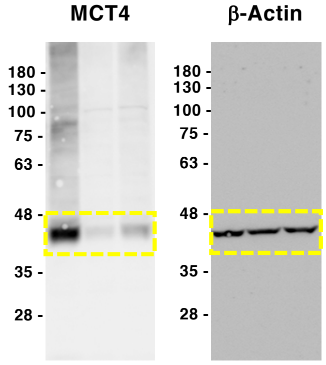

Fig. 4f

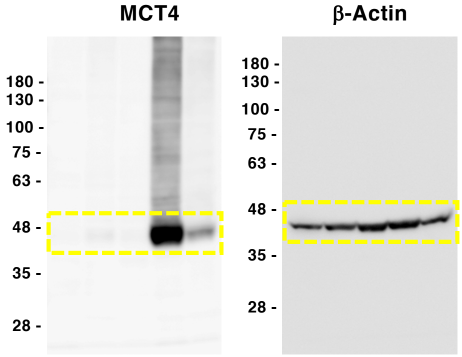

Fig. 6b

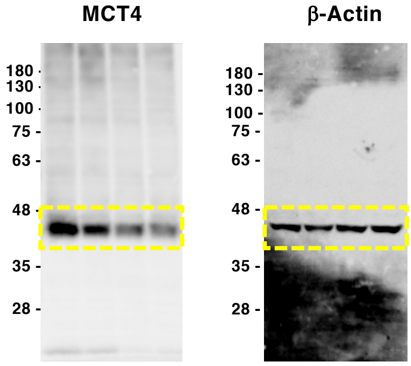

Fig. 6c

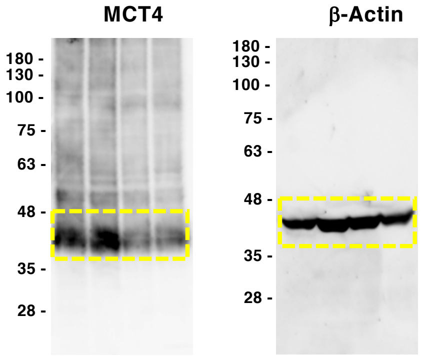

Fig. 6e

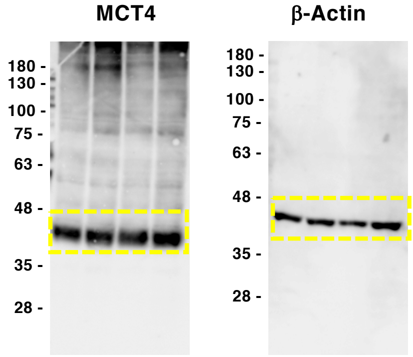

Fig. 6f

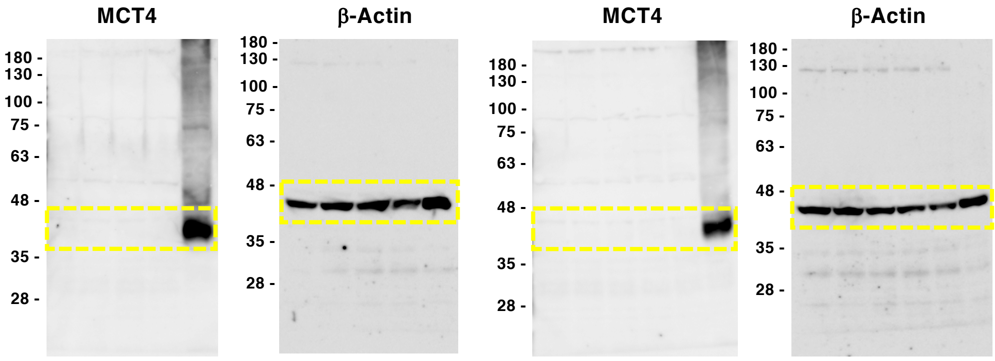

Fig. 6f

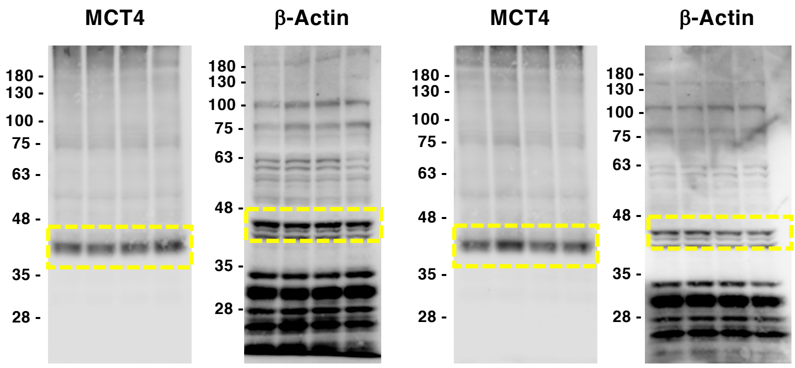

Fig. 7a

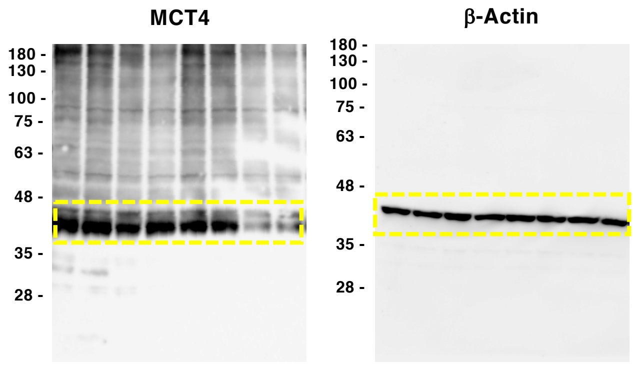

Fig. 7b

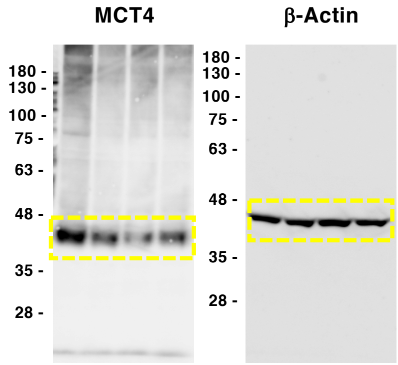

Fig. 7c

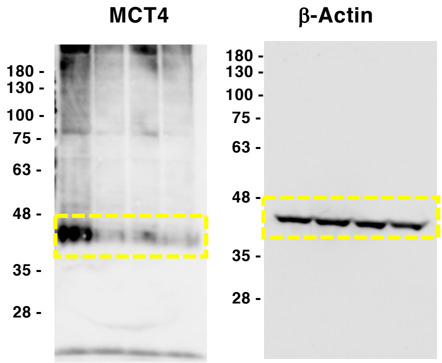

Fig. 7d

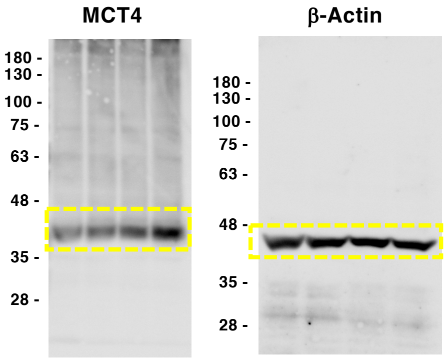

Fig. 7f

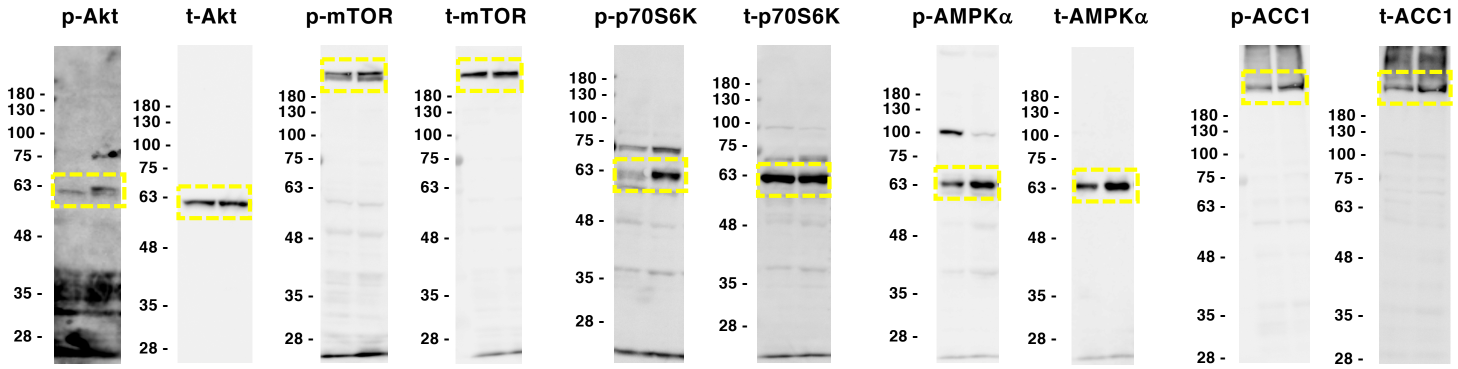

Fig. 7f

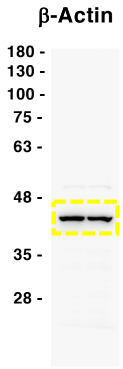

Fig. 7g

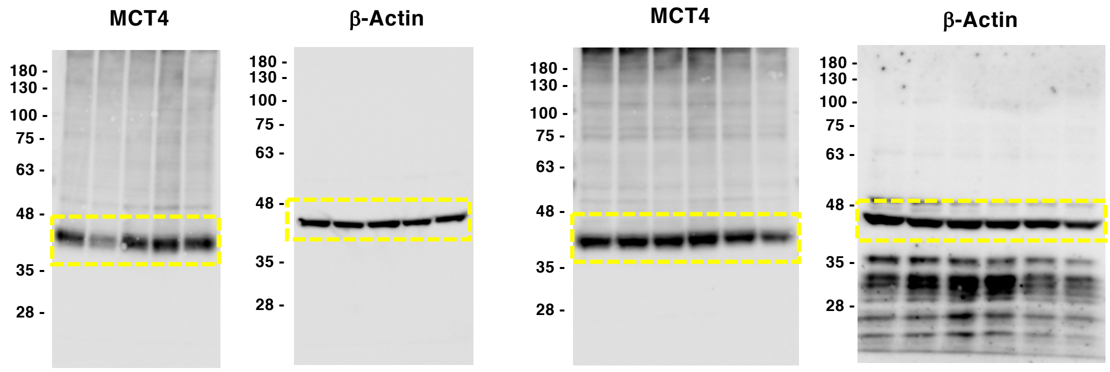

Fig. S9a

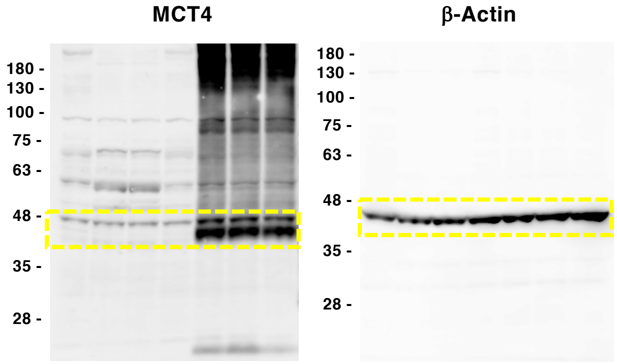

Fig. S9b

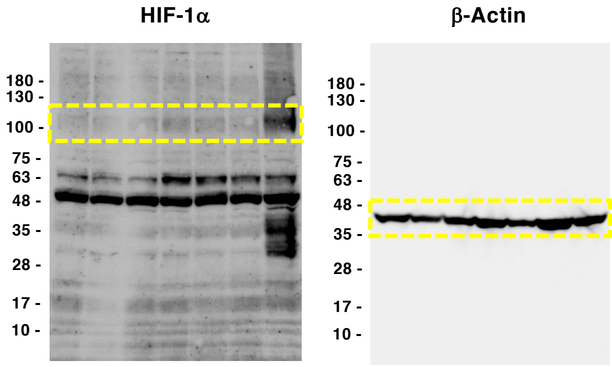

Fig. S9c

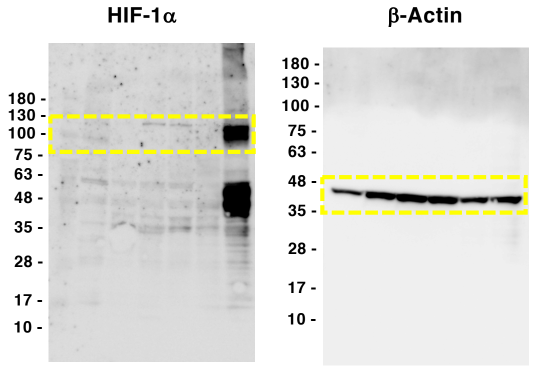

Fig. S10a

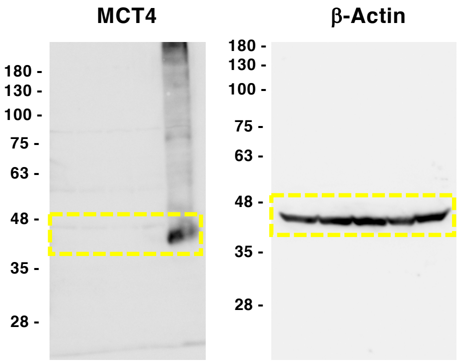

Fig. S10b

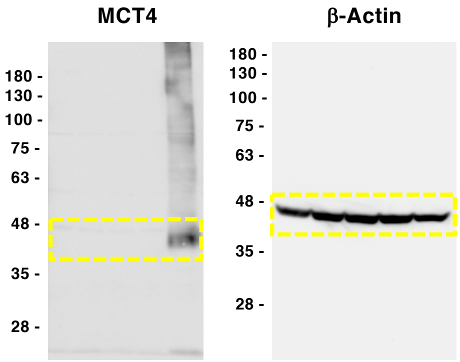

Fig. S10c

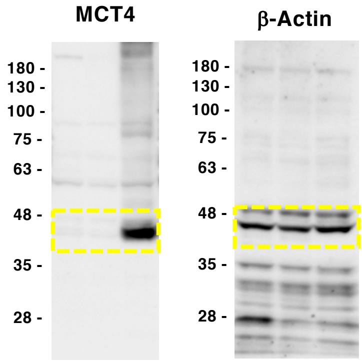

Supplementary Fig. S12. Full-size images of Western blots. The yellow dotted line indicates the cropped region.
